# Social cognition and decision‐making in people with methamphetamine use disorder

**DOI:** 10.1111/add.70108

**Published:** 2025-06-16

**Authors:** Justin Mahlberg, Lauren Hanegraaf, Josua Zimmermann, David M. Cole, Boris B. Quednow, Shalini Arunogiri, Antonio Verdejo‐Garcia

**Affiliations:** ^1^ School of Psychological Sciences and Turner Institute for Brain and Mental Health Monash University Clayton Australia; ^2^ Experimental Pharmacopsychology and Psychological Addiction Research, Department of Adult Psychiatry and Psychotherapy University Hospital of Psychiatry Zurich, University of Zurich Zurich Switzerland; ^3^ Neuroscience Center Zurich University of Zurich and Swiss Federal Institute of Technology Zurich Switzerland; ^4^ Translational Psychiatry Lab University Psychiatric Clinics Basel, University of Basel Basel Switzerland; ^5^ Monash Addiction Research Centre Eastern Health Clinical School, Faculty of Medicine, Nursing and Health Sciences, Monash University Melbourne Australia; ^6^ Turning Point Eastern Health Melbourne Australia

**Keywords:** aggression, decision‐making, emotions, empathy, methamphetamine, social cognition, trust

## Abstract

**Background and Aims:**

Impairments in social cognition and social decision‐making play an important role in the disease burden experienced by individuals with methamphetamine use disorder (MUD). They are also assumed to play a role in the vicious cycle of MUD development and hinder its successful psychotherapy. However, research typically focuses on examining specific types of social cognitive deficits in MUD, rather than profiling the multidimensional social cognition and decision‐making impairments that coincide with MUD. Our study, thus, estimated the socio‐cognitive and social decision‐making profile of people with MUD and compared this profile with a methamphetamine‐naïve control group.

**Design:**

Cross‐sectional case–control comparison of social cognition and decision‐making between participants with MUD recruited from clinical services and methamphetamine‐naïve controls (CTRL) recruited from the community.

**Setting:**

Drug treatment clinics in Melbourne, Australia.

**Participants:**

52 participants with MUD (moderate or severe; 77% identified as male) between April 2019 and September 2021 and 51 demographically matched CTRLs (no history of methamphetamine use; 54% identified as male) between May and September of 2021.

**Measurements:**

We implemented a social cognition battery that assessed a participant's sensitivity for perceiving emotion from faces (emotion recognition) and their ability to detect accurately the emotional state of others (cognitive empathy) and experience the emotions of others (emotional empathy). We also characterised the propensity to engage in higher‐order social decision‐making by assessing a participant's willingness to engage in interpersonal trust and aggression in experimental simulations.

**Findings:**

Compared with matched controls, people with MUD had a bias toward perceiving happier facial expressions as neutral [Estimate = −5.05, standard error (SE) = 2.05, *P* = 0.016, 95% confidence interval (95% CI) = (−9.12 to −0.98)], showing lower sensitivity to perceiving happy emotions [Estimate = −6.34, SE = 3.09, *P* = 0.043, 95% CI = (−12.47 to −0.20)]. People with MUD also showed a propensity to enact more intense punishments [Estimate = 1.48, SE = 0.38, *P* < 0.001, 95% CI = (0.73–2.23)] and lower levels of trust to others in their decisions [Estimate = −0.11, SE = 0.04, *P* = 0.002, 95% CI = (−0.18 to −0.04)].

**Conclusions:**

In a clinical research study, people with methamphetamine use disorder appeared to show lower sensitivity to happy emotions, reduced trust and increased aggression toward others, relative to a matched control group.

## INTRODUCTION

Methamphetamine use disorder (MUD) is associated with dysfunction across numerous interpersonal domains [1], including aggression [2, 3]. This may be underpinned by difficulties with social cognition (SC), which is the ability to perceive, understand and respond to the dispositions and behaviours of others [4]. Difficulties with SC are thought to increase social isolation, aggression and depression, likely predisposing toward substance use [2]. Chronic methamphetamine use may further degrade SC abilities [5]. Increased SC difficulties may contribute to interpersonal dysfunction, escalate the vicious circle of MUD development and impede successful psychotherapeutic interventions [5]. Developing a nuanced understanding of the SC profile of people with MUD may be critical for improving social and clinical outcomes.

SC encompasses abilities critical for social interaction [6]. Emotion recognition (ER) refers to the ability to perceive others' emotions [7], while empathy comprises cognitive (i.e. understanding the emotions of others) and emotional (i.e. experiencing the emotions of others) components [8]. Broadly, substance use disorders coincide with blunting of empathy in addition to lower‐level emotion recognition deficits [9, 10, 11]. People with MUD exhibit poorer performance on cognitive empathy tasks [reading‐the‐mind‐in‐the‐eye‐test (RMET)] [7, 12, 13], and both broad and emotion‐specific (e.g. anger) ER deficits [13]. However, this prior evidence assessed accurate categorisation of emotional expressions, and it is unclear if deficits are the result of an under‐ or over‐sensitivity to emotional cues. A recent study found no evidence of differences between MUD and control groups when assessing sensitivity to emotions [14]. Moreover, Zacher *et al*. [14] assessed both cognitive and emotional empathy and found that the response to positive stimuli was diminished, while empathy for negative stimuli remained similar to controls. This finding is surprising because prior evidence has commonly shown ER deficits in MUD for negative emotions [7, 13, 15], however, prior studies have questioned the psychometrics of the RMET, which could explain these inconsistencies [16, 17].

SC dysfunction may also be important for predicting decisions to act aggressively or trust another [18]. These behaviours are of interest as they potentially contribute to MUD‐related burden of disease and treatment outcome [5]. The decision to trust or behave aggressively toward others involves both anticipated costs and benefits [19] and is associated with the recruitment of reward [20] and central‐executive [21] neural networks. Chronic methamphetamine use coincides with functional deficits in these networks [22] and has been linked to an uptick in aggressive behaviour in animal studies [23]. Despite this, research investigating these behaviours in MUD is scarce. There are no studies measuring people with MUD's propensity to engage in interpersonal trust, although self‐report studies indicate the tendency to extend interpersonal trust covaries with aggression [24], and people with MUD acted less prosocial than healthy controls in a modified dictators game [25]. Two studies have captured an objective measure of aggression in people with MUD: in a competitive reaction time task (CRTT), people with MUD showed a heightened tendency for acting aggressively—overall and when provoked through receiving punishment [14, 26].

We aimed to characterise SC and social decision‐making in people with MUD. We tested participants with MUD on a SC battery assessing sensitivity to perceiving emotions (i.e. ER), detecting the emotions of others (cognitive empathy) and experience of another's emotions (emotional empathy). We also assessed willingness to engage in interpersonal trust and aggression using experimental simulations. We expected that individuals with MUD, relative to healthy controls, would exhibit differences in SC abilities (i.e. reduced capacity for empathy and ER) and social decision‐making behaviours (i.e. increased aggression, reduced trust). We additionally explored the relationship between SC, social decision‐making and clinical characteristics in our sample.

## METHOD

### Design

Cross‐sectional case–control comparison of SC and social decision‐making between participants with MUD and methamphetamine‐naïve controls (CTRL).

### Participants

Participants were recruited in Melbourne, Australia. The CTRL were recruited via community advertisements to match the MUD group on socio‐demographic variables that may influence SC, including age, education and verbal intelligence quotient (IQ).

People with MUD were eligible if they demonstrated a primary diagnosis of MUD, using the Structured Clinical Interview for the Diagnostic and Statistical Manual of Mental Disorders, fifth edition (DSM‐5) [27], administered by trained study investigators. Participants with conditions impacting the central nervous system were excluded. Controls were also excluded if they reported lifetime methamphetamine use or risky alcohol use based on Australian national health guidelines [28].

### Computerised SC measures

#### Emotion recognition: Face morphing task

Face morphing task (FMT) [14, 29] trials showed a face that could be morphed dynamically via horizontal mouse movements across 100 frames ranging from happy to angry (scored 0%–100%), with neutral positioned in the middle (50%). Emotion sensitivity was evaluated in three conditions by asking participants to find the most neutral face, the first sign of anger or the first sign of happiness in each trial. Each condition included 13 trials, and their presentation was randomised across participants.

#### Empathy: Multifaceted empathy test

Participants were presented with 40 pictures of demographically diverse individuals in emotionally charged situations. Images were rated separately for cognitive and emotional empathy and were randomly presented in four blocks of 10 images [30, 31]. Cognitive empathy was assessed by asking participants to select the correct emotional label from four choices. Emotional empathy was assessed by asking participants to rate their level of empathy for the person in the image on a 1 to 9 scale (1 = not at all, 9 = very much).

### Social decision‐making measures

#### Interpersonal trust: Trust game

Participants were instructed to collaborate with another player (the investor), which is—unbeknown to the participant—simulated by an adaptive algorithm that draws responses from a dataset of human players [32] (see Supporting information for details). Across each of the 10 rounds, the investor was given $20 of digital currency and decided how much to keep and how much to invest. The amount invested was multiplied by three and given to the participant. The trustee (the participant) then decided at the end of each round how much of these returns they would give back to the investor. Participants were instructed that after the task, they could claim a reimbursement based on a scaled version of the total amount of money earned.

#### Aggression: CRTT

Across 25 CRTT [33] trials, participants needed to outperform a simulated opponent by pressing a key quicker during interactions designed to assess provoked and non‐provoked punishment responses. Participants were instructed that the opponent player was a different person than who played the trust game. Before each trial, players set the punishment intensity their opponent would receive if they lost. The punishment was noise, delivered via headphones, ranging between eight intensity levels. The outcome (win/loss) of each trial depended on the participant's reaction time and the outcome of the previous trials, and the punishment settings of the opponent followed a pseudo‐randomised order.

### Demographic and clinical measures

Socio‐demographic variables (e.g. age, gender and education) were collected via an *ad hoc* survey and verbal IQ was estimated using the National Adult Reading test [34]. We used well‐validated tools to characterise methamphetamine use patterns [Timeline Followback (TLFB)] [35] and severity [Severity of Dependence Scale (SDS)] [36], early adverse experiences [Adverse Childhood Experiences Scale (ACES)] [37] and quality of life (World Health Organisation Brief Quality of Life scale) [38]. We additionally assessed co‐occurring depression [Center for Epidemiologic Studies Depression Scale–Revised (CESD‐R)] [39], anger expression (State–Trait Anger Expression Inventory) [40] and psychotic experiences (Community Assessment of Psychic Experiences) [41].

### Procedure

All participants deemed eligible after a phone screen were tested face‐to‐face, except for 12 CTRL participants who were assessed via the video‐conferencing platform Zoom using control screen technology because of coronavirus disease 2019 lockdowns. The assessment battery order was standardised across participants using well‐validated guidelines [42]. The battery took approximately 2 hours for the MUD group, and approximately 1.5 hours for the CTRL group. All participants provided informed consent, and this study was approved by the Human Research Ethics Committee at Monash University (ID: 5943).

### Statistical analysis

The analysis was not pre‐registered and the results should be considered exploratory. All data analysis was conducted using R version 4.3.2. Cognitive data were fit with multilevel models. Full model details and sensitivity analyses are reported in the Supporting information (see Tables S1–S9). Complete cases were required for the cognitive data. For covariates and clinical data, missing values were handled using pairwise deletion. Table 1 reports MUD characteristics. Group comparisons reported in Table 2 were two‐sample *t* tests when data approximated normality and groups had equal variance. Welch's *t* test was used when group variance was unequal (assessed with Levene's test), and Wilcox's rank sum was calculated when data was non‐normal. All *P* values are significant at 0.05. In all task analysis [except for multifaceted empathy test (MET) emotional empathy and CRTT], data was maintained as individual trial‐level observations and within‐subject variance was controlled with random effects. For MET emotional empathy and CRTT, trial‐level observations deviated from normality and impacted model fit, therefore we summarised responses to the level of the fixed and random effects (see Supporting information for a detailed description). Random effects models were selected by fitting the maximal unconditional random effects [43] to account for known sources of variation. In cases where maximal random effect structures did not converge, random effects were iteratively simplified by removing variables that explained near zero variance or removing slope effects. All distribution model assumptions were assessed by visual inspection of plots (e.g. residual and density plots) produced with the *performance* package (see Figures S1–S5 [44]).

**TABLE 1:** Clinical profile of the participants with MUD.

MUD characteristics	*n*	M or count	(SD) or %
Severity of dependence scale	52	8.83	(3.31)
Timeline follow back (days)	52	17.52	(9.20)
DSM‐5 symptom count	52	8.87	(2.10)
Mild (2–3 symptoms)	52	1	1.92%
Moderate (4–5 symptoms)	52	4	7.69%
Severe (6+ symptoms)	52	47	90.39%
Age of onset (y)	50	30.74	(11.21)
Disorder duration (y)	49	7.43	(7.29)
Urine drug screen			
Methamphetamines	50	41	82.00%
Amphetamines	50	35	70.00%
Benzodiazepines	48	18	37.50%
Cocaine	50	2	4.00%
THC	50	11	22.00%
MDMA	47	5	10.64%
Opioid	49	8	16.33%
Current psychotic disorder (drug induced)	51	12	23.53%

*n =* sample size for the measure; values in second column from the right represent means (M) or frequency (*c*ount); the right most column indicates standard deviation of the mean (SD) or proportion of *n* (%).

DSM‐5, Diagnostic and Statistical Manual of Mental Disorders, fifth edition; MDMA, 3,4‐methyl enedioxy methamphetamine; MUD, methamphetamine use disorder; THC, Δ‐9‐tetrahydrocannabinol.

**TABLE 2:** Participant characteristics for each group.

	*n*	MUD		*n*	CTRL		Test	Sig.
Age	51	38.22	(9.34)	50	38.52	(13.81)	0.13^a^	0.90
Self‐identified gender (% male)	51	39	76.47%	48	26	54.17%	2.30^b^	0.021
Education (y)	49	13.08	(2.89)	50	14.32	(3.25)	2.00	0.048
Estimated IQ (NART scores)	51	111.21	(8.94)	50	111.83	(7.28)	0.38	0.71
Quality of life (WHOQOL‐BREF)								
Total	52	73.71	(16.65)	50	104.58	(12.79)	10.47	<0.001
Physical	52	22.88	(5.54)	50	28.90	(4.16)	6.22^a^	<0.001
Psychological	52	17.31	(5.04)	50	22.48	(3.54)	6.02^a^	<0.001
Social	52	8.27	(2.55)	50	11.64	(2.42)	6.84	<0.001
Environmental	52	25.25	(5.61)	50	33.88	(4.42)	8.60	<0.001
Depression (CES‐D)	52	27.69	(16.04)	50	21.42	(15.02)	1627^c^	0.029
Psychotic experiences (CAPE‐15)	52			50				
Total	52	22.81	(6.50)	50	17.52	(2.79)	2070^c^	<0.001
Persecutory ideation	52	9.56	(2.80)	50	6.82	(1.75)	5.94^a^	<0.001
Perceptual abnormalities	52	4.27	(1.74)	50	4.06	(0.24)	0.84	0.40
Bizarre experiences	52	8.98	(3.02)	50	7.66	(1.30)	1644.50^c^	0.01
Adverse childhood experiences (ACES)	52	3.75	(2.88)	50	2.94	(2.71)	1506^c^	0.17
Anger expression index (STAXI)	52	36.90	(13.11)	50	26.82	(12.07)	4.04	<0.001

Data for each group (MUD, CTRL) represent mean (SD) for each measure, except for gender, which shows frequency of participants who reported a male gender and the proportion of males in each group. *n* = the data available for each group for each measure. Test reports the statistical test applied for each measure to test group differences. Two‐sample *t* tests were applied unless marked otherwise. Sig. indicates the significance test for each measure. Tests were significant when < 0.05.

ACES, Adverse Childhood Experiences Scale; CAPE‐15, Community Assessment of Psychic Experiences, 15 items; CES‐D, Center for Epidemiologic Studies Depression Scale; CTRL, methamphetamine‐naïve controls; MUD, methamphetamine use disorder; NART, National Adult Reading Test; STAXI, State–Trait Anger Expression Inventory; WHOQOL‐BREF, World Health Organisation Brief Quality of Life scale.

^a^ Indicates Welch's two‐sample *t* was used because of unequal variances.

^b^ Indicates a logistic regression was applied to test gender, and a z test is reported.

^c^ Indicates Wilcox's rank sum was used because of non‐normality. Sig. indicates the significance test for each measure. Tests were significant when <0.05.

Statistical models for all tasks included gender and education as covariates. The ER model assessed the effects of condition (happy, neutral or angry face), group (MUD, CTRL) and their interaction. Group contrasts evaluated the hypothesis that groups differed in their sensitivity to happy, neutral and angry emotions. Empathy processing was tested by fitting (a) a binomial generalised linear mixed effects model with a logit link to assess the likelihood of accurate cognitive empathy responses; and (b) a linear mixed effects model assessing emotional empathy. Each model evaluated the hypothesis that empathy (cognitive and emotional, respectively) varied by group (MUD, CTRL), and emotion type (positive vs. negative) impacted empathy in the MUD group. Trust was evaluated using the proportion of the total returns given to the investor as a function of group (MUD, CTRL) to test the hypothesis that people with MUD show reduced interpersonal trust. Aggression was examined via the punishment intensity set by participants as a function of group (MUD, CTRL) and whether the participant experienced punishment on the prior trial opponent, which tested the hypotheses that people with MUD are more aggressive in general and are more reactive in their aggression (i.e. are more aggressive after receiving punishment). All hypothesis tests—and for completeness all fixed effects—are reported in Table 3. We also explored how the SC and decision‐making battery overlapped with clinically related addiction and psychosocial characteristics using Spearman's ρ, adjusted for the false discovery rate [45], to assess the correlation between all tasks and clinical measures.

**TABLE 3:** Results for all model analyses of the social cognition and decision‐making tasks.

Model	Predictor	d.f.	Estimate	SE	Sig.	95% CI	Eff. size
FMT^a^	Gender [male]	90.00	0.71	1.63	0.66	−2.53 to 3.95	0.03
	Education	90.00	−0.05	0.25	0.84	−0.54 to 0.44	−0.01
	Group [MUD]	94.01	−5.05	2.05	0.016	−9.12 to −0.98	−0.21
	Condition [happy]	92.00	−14.34	2.10	<0.001	−18.50 to −10.18	−0.59
	Condition [angry]	91.99	18.38	2.45	<0.001	13.51–23.25	0.76
	Condition [happy] × group [MUD]	92.00	−1.28	2.96	0.67	−7.17 to 4.60	−0.05
	Condition [happy] × group [angry]	91.99	10.28	3.46	0.004	3.40–17.16	0.42
Neutral^b^	Group [MUD vs. CTRL]	94.0	−5.05	2.05	0.016	−9.12 to −0.98	−0.38
Happy^b^	Group [MUD vs. CTRL]	94.8	−6.34	3.09	0.043	−12.47 to −0.20	−0.48
Angry^b^	Group [MUD vs. CTRL]	94.9	5.23	3.09	0.094	−0.90 to 11.36	0.40
MET cognitive^c^	Gender [male]	–	−0.008	0.12	0.94	0.79–1.24	0.99
	Education	–	0.02	0.02	0.159	0.99–1.06	1.02
	Group [MUD]	–	0.07	0.13	0.588	0.83–1.39	1.07
	Valence [positive]	–	0.65	0.10	<0.001	1.57–2.33	1.91
	Valence [positive] × group [MUD]	–	−0.13	0.15	0.379	0.65–1.18	0.88
MET emotional^a^	Gender [male]	82.00	0.23	0.39	0.56	−0.55 to 1.01	0.11
	Education	82.00	−0.03	0.06	0.59	−0.15 to 0.08	−0.05
	Group [MUD]	84.34	0.52	0.47	0.27	−0.41 to 1.45	0.25
	Valence [positive]	84.00	0.21	0.32	0.50	−0.42 to 0.85	0.10
	Valence [positive] × group [MUD]	84.00	−0.89	0.48	0.07	−1.84 to 0.06	−0.43
MUD^b^	Valence [positive vs. negative]	84.00	−0.68	0.36	0.06	−1.39 to 0.03	−0.91
CTRL^b^	Valence [positive vs. negative]	84.00	0.22	0.32	0.50	−0.42 to 0.85	0.29
Trust game^a^	Gender [male]	89.65	0.02	0.04	0.65	−0.06 to 0.09	0.07
	Education	89.18	0.005	0.01	0.42	−0.01 to 0.02	0.06
	Group [MUD]	89.58	−0.11	0.04	0.002	−0.18 to −0.04	−0.46
CRTT^a^	Gender [male]	91.00	0.06	0.39	0.88	−0.71 to 0.82	0.03
	Education	91.00	−0.05	0.06	0.37	−0.17 to 0.06	−0.09
	Group [MUD]	99.27	1.48	0.38	<0.001	0.73–2.23	0.77
	Condition [lost prior trial]	93.00	0.16	0.11	0.144	−0.06 to 0.38	0.09
	Condition [lost prior trial] × group [MUD]	93.00	−0.07	0.16	0.64	−0.39 to 0.24	−0.04

95% CI, 95% confidence interval for the estimate; CRTT, competitive reaction time task; CTRL, methamphetamine‐naïve controls; d.f., degrees of freedom; Eff. Size, standardised effect size; Estimate, the coefficient for the fixed effect; FMT, face morphing task; MET, multifaceted empathy test; MUD, methamphetamine use disorder; Predictor, the effects tested; SE, standard error for the estimate; Sig., significance test (*P* value).

^a^ Indicates effects were produced with a linear mixed effects model using Satterthwaite estimation for d.f. and significance tests. These model effects, effect sizes reported are standardised β.

^b^ Indicates contrasts from model estimated marginal means, and effect sizes reported are Cohen's D.

^c^ Indicates effects were produced with a logistic mixed effects model and significance tests were estimates using Wald's Z‐test and effect sizes reported are ORs.

## RESULTS

### Participants characteristics

People with MUD (*n* = 121) were contacted via referrals from Turning Point and other drug treatment services and community and social media advertising in Melbourne, Australia between April 2019 and September 2021. A total of 95 met pre‐specified eligibility criteria and were invited to participate, and 52 completed the study. Nine MUD participants had missing data for the MET, three MUD participants had missing trust game data and the FMT and CRTT were both missing one MUD participant. Most participants with MUD (90%) experienced severe substance use disorder (6+ symptoms) (Table 1) and an average disorder duration of 7.43 years (SD = 7.29). Methamphetamine use was recent and regular for most participants according to TLFB reports and urinalysis. Substance‐induced psychosis rates were consistent with prevalence rates in prior Australian samples [46].

CTRL participants (*n* = 51) were recruited between May and September in 2021, and one was excluded because of recent methamphetamine use. One CTRL was missing FMT data. The remaining (*n* = 50) were matched on age and estimated verbal IQ, although Table 2 shows group differences were observed as more MUD participants were men compared to the CTRL group and had a slightly lower education. The MUD group showed higher depression severity, substantially lower quality of life, higher rates of persecutory ideation and bizarre experiences and higher state anger expression compared to the CTRL group.

### Groups differences across social cognitive measures

#### Emotion recognition (FMT)

**FIGURE 1:**
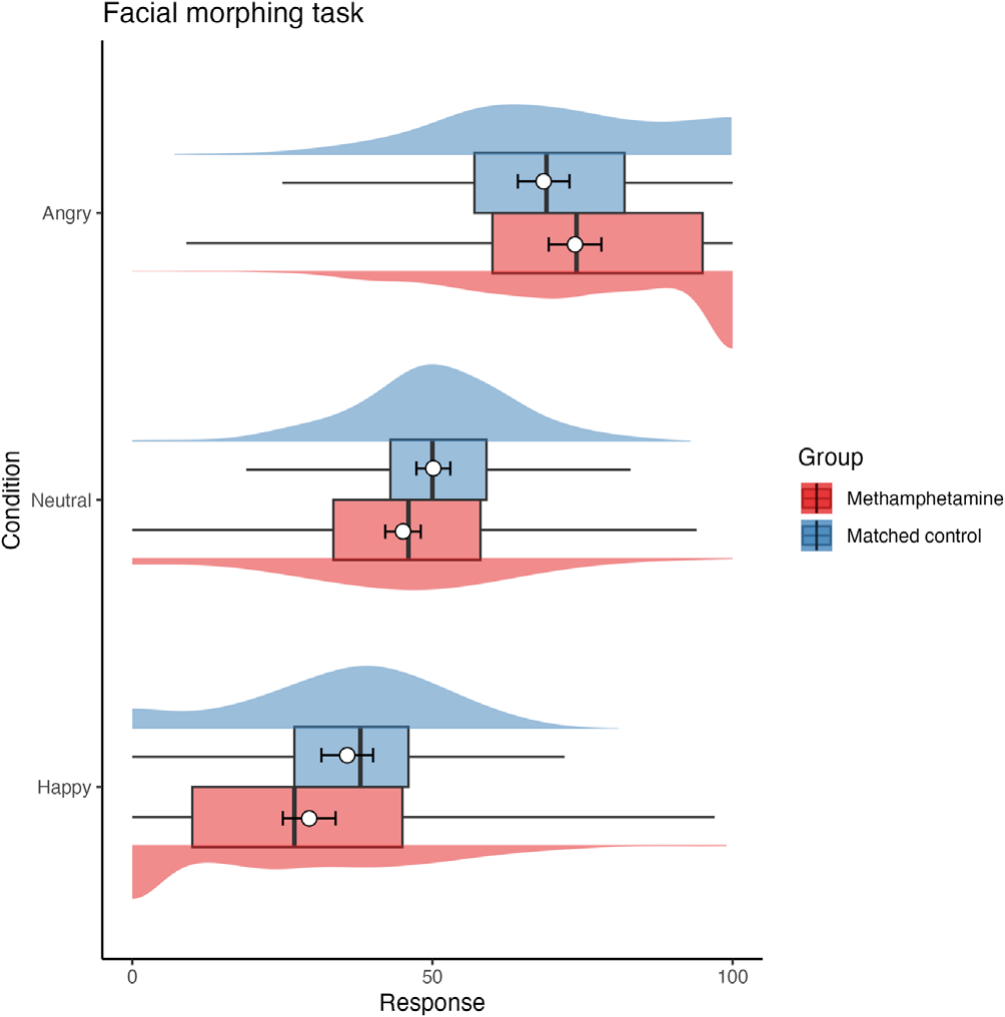
Responses for each group and condition in the facial morphing task. The response variable is plotted on the X axis, and conditions (angry, neutral, happy) are plotted on the Y axis. A response of 50 is considered a neutral emotional expression. A response of 100 would indicate a face with the maximum level of anger was selected, whereas a response of 0 would indicate a face with the maximum level of happiness was selected. The data plotted on the top for each condition show the methamphetamine use disorder (MUD) group in red, while the data plotted on the bottom for each condition show the matched control group. The box plots show the median and interquartile range of the distribution. The adjacent half‐eye plots show the distribution of the responses for each group and condition. The white circles plot the model estimated marginal means and the error bars plot the 95% CI.

Estimates for FMT are shown in Figure 1 and Table 3. There was no evidence that gender (*P* = 0.66) or education (*P* = 0.84) impacted responses. Nevertheless, after adjusting for these characteristics, the MUD group showed a significant bias toward rating faces that retain more happy features as neutral compared to the CTRL group (*P* = 0.016) and indicated the first sign of happy faces later than the CTRL group (*P* = 0.043) whereas groups were similar for angry faces (*P* = 0.094).

#### Empathy processing (MET)

**FIGURE 2:**
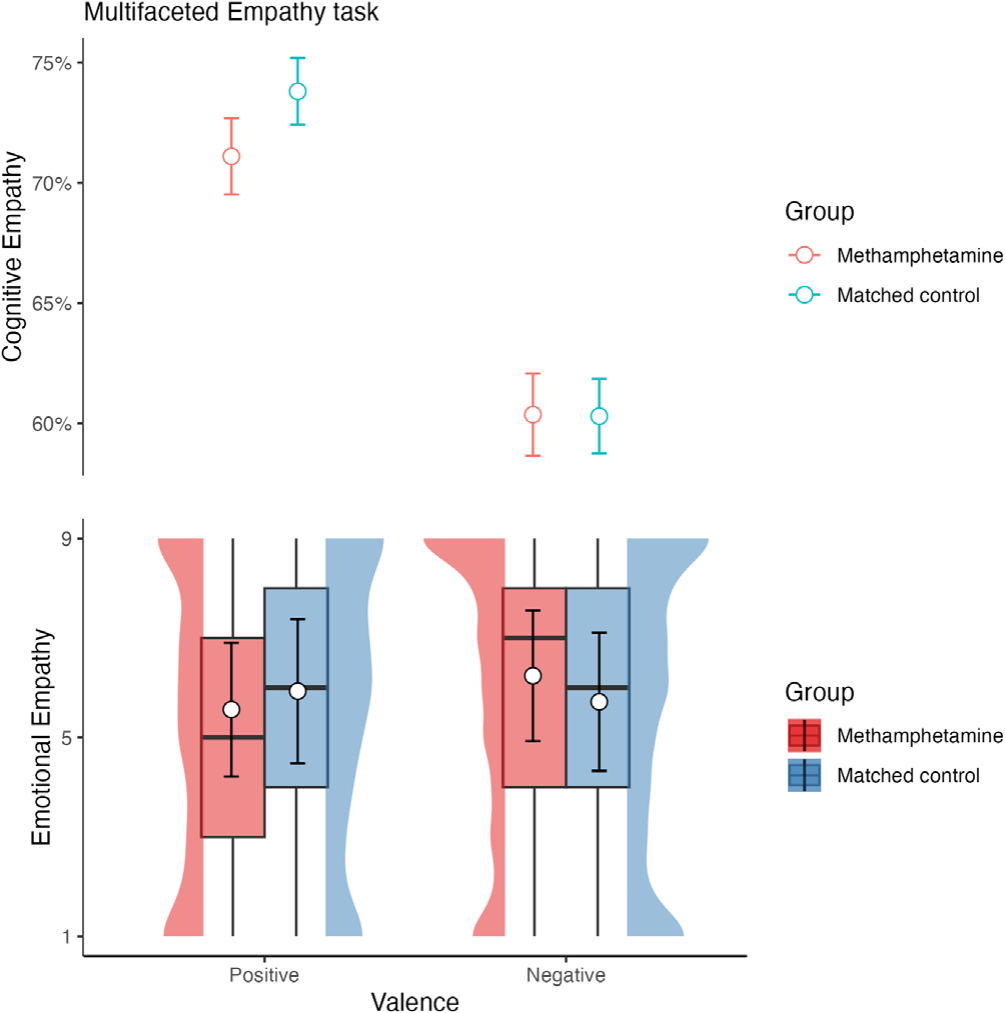
Responses for each group and condition in the multifaceted empathy task. The top panel plots the proportion of correct responses in the cognitive empathy condition on the y axis by positive and negative valence trials. The bottom panel plots the emotional empathy responses by positive and negative valence trials. The data plotted to on the left for each condition show the methamphetamine use disorder (MUD) group in red, while the data plotted on the right for each condition show the methamphetamine‐naïve controls (CTRL) group in blue. The box plots show the median and interquartile range of the distribution. The adjacent half‐eye plots show the distribution of the responses for each group and condition. The white circles plot the model estimated marginal means and the error bars plot the 95% CI.

Figure 2a plots the accuracy for cognitive empathy, and Figure 2b plots the extent of emotional empathy. As Table 3 shows, there was no evidence that cognitive empathy was impacted by gender (*P* = 0.94) or education (*P* = 0.16). Overall, participants were better at detecting positive compared to negative emotions (*P* < 0.001). There was no evidence that group (*P* = 0.59) or the interaction between group and valence, *P* = 0.38, predicted cognitive empathy. There was no evidence that gender (*P* = 0.56) or education (*P* = 0.59) impacted emotional empathy or that either valence (*P* = 0.50) or group (*P* = 0.27) impacted emotional empathy. MUD participants were marginally less sensitive to positive stimuli (*P* = 0.06), whereas the CRTL group showed no differences across stimulus valence (*P* = 0.50).

#### Interpersonal trust

**FIGURE 3:**
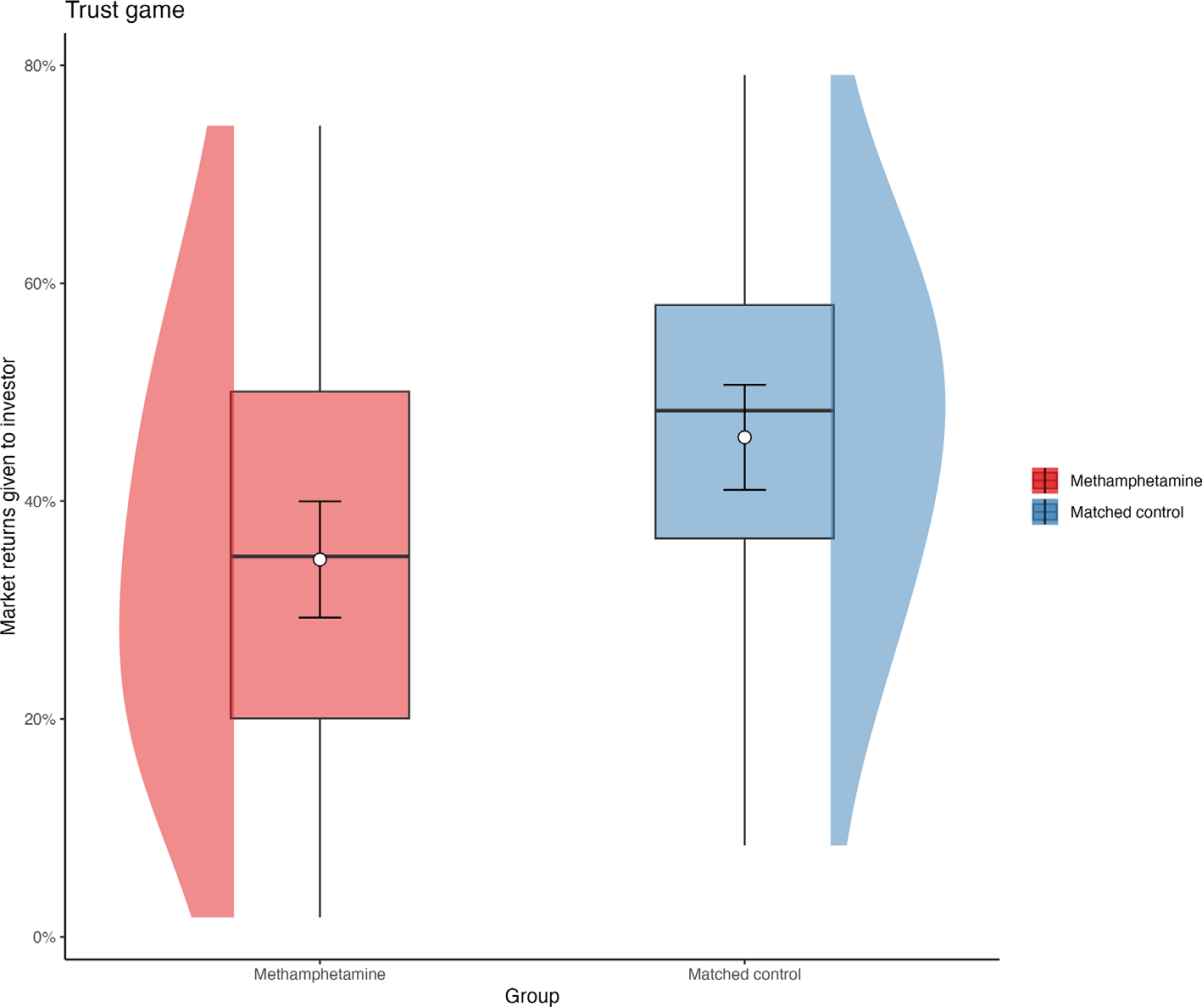
Proportion of the total market returns participants received and returned to the investor for each group in the trust game. The data plotted to on the left show the methamphetamine use disorder (MUD) group in red, while the data plotted on the right for each condition show the methamphetamine‐naïve controls (CTRL) group in blue. The box plots show the median and interquartile range of the distribution. The adjacent half‐eye plots show the distribution of the responses for each group and condition. The white circles plot the model estimated marginal means and the error bars plot the 95% CI.

There was no evidence that gender (*P* = 0.65) and education (*P* = 0.42) significantly predicted trust responses. As Figure 3 and Table 3 indicates, MUD entrusted a significantly lower proportion of the returns to the investor compared to the CTRL group (*P* = 0.002).

#### Aggression (CRTT)

**FIGURE 4:**
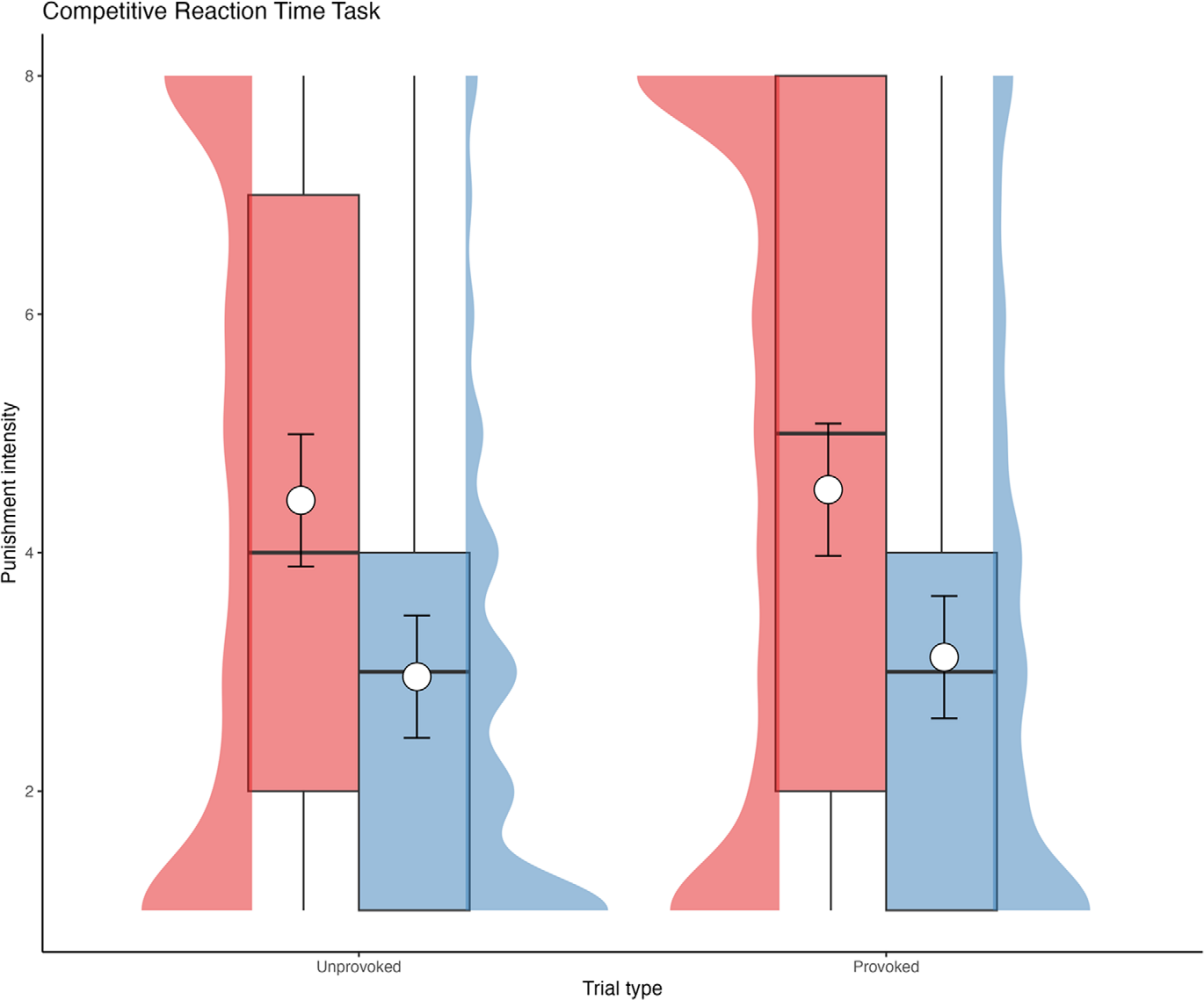
Punishment intensity settings for each group for trials after they were punished on the previous trial (retaliatory) and trials where they were not punished on the previous trial (non‐retaliatory). The data plotted on the left for each condition show the methamphetamine use disorder (MUD) group in red, while the data plotted on the right for each condition show the methamphetamine‐naïve controls (CTRL) group in blue. The box plots show the median and interquartile range of the distribution. The adjacent half‐eye plots show the distribution of the responses for each group and condition. The white circles plot the model estimated marginal means and the error bars plot the 95% CI.

Figure 4 and Table 3 shows there was no evidence that aggression varied as a function of gender (*P* = 0.88) or education (*P* = 0.37). The MUD group selected more intense punishments for their opponent compared to the CTRL group (*P* < 0.001). There was no evidence of retaliation, insofar as punishment intensity was similar whether or not punishments were experienced on the prior trial (*P* = 0.14) or for an interaction with group (*P* = 0.64).

### Association between clinical characteristics and social‐cognitive tasks

**FIGURE 5:**
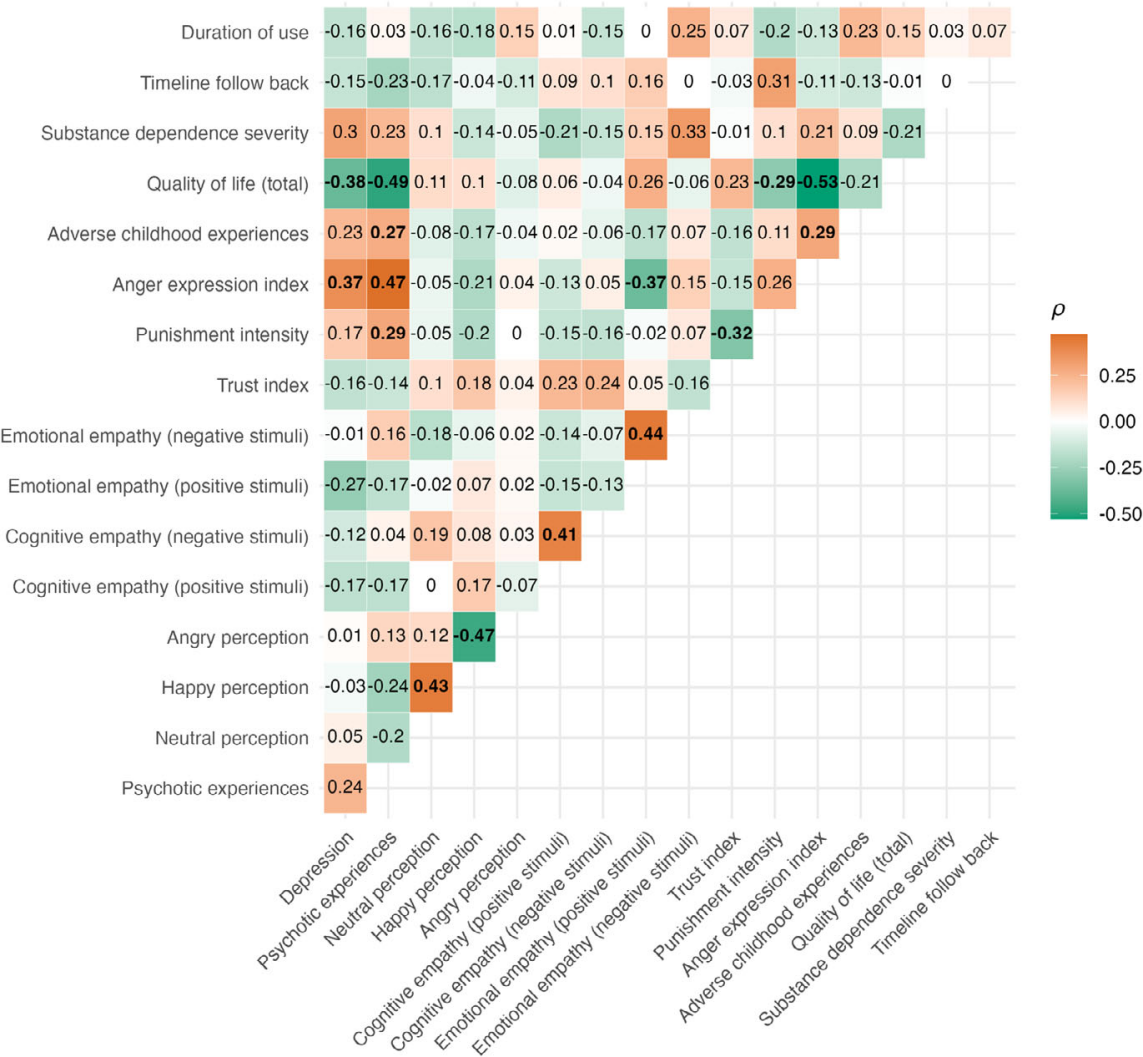
A correlation matrix showing the relationships between the task measures and psychosocial measures. Where data were available for both groups (e.g. psychosocial measures), all data were used to compute relationships, whereas correlations that included drug‐specific measures only involved the methamphetamine use disorder (MUD) group. Correlations are calculated with Spearman's ρ, and statistical significance was adjusted using the false discovery rate adjustment. After adjustment, coefficients that are *P* < 0.05 are boldfaced.

All measures showed optimal psychometric performance (Cronbach's α range = 0.73–0.97). Figure 5 shows the full correlation matrix. Emotional empathy for positive stimuli correlated negatively with anger expression [ρ = −0.37, *P* = 0.005, 95% CI = (−0.53 to −0.19)]. Trust game scores negatively correlated with the intensity of CRTT punishments [ρ = − 0.32, *P* = 0.02, 95% CI = (−0.48 to −0.14)]. The intensity of CRTT punishments also positively correlated with psychotic experiences [ρ = 0.29, *P* = 0.03, 95% CI = (0.10–0.46)] and negatively correlated with quality of life [ρ = −0.29, *P* = 0.03, 95% CI = (−0.46 to −0.10)]. All other correlations were non‐significant (*P* > 0.05).

## DISCUSSION

People with MUD exhibited dysfunction in SC and social decision‐making. Specifically, relative to controls, they showed reduced sensitivity in recognising happy emotions, reduced interpersonal trust and an increased propensity to enact more intense punishments to others. However, both cognitive and emotional empathy abilities were broadly comparable across our groups. Altogether, our findings suggest that MUD is associated with impairments in both lower‐level SC and higher‐order social decision‐making.

Our ER findings may help clarify whether ER inaccuracy in people with MUD reflects hypersensitivity or hyposensitivity in emotion recognition. Our evidence shows people with MUD require more perceptual evidence to classify faces as happy, which indicates hyposensitivity to happy facial expressions. This resulted in MUD participants requiring stronger happy facial expressions to identify happiness and perceiving faces with some mild happy facial expressions as neutral. This adds nuance to a prevailing view that ER deficits associated with MUD may contribute to misinterpretation of threat cues, resulting in individuals responding pre‐emptively in an aggressive manner to benign social stimuli [7, 13, 15]. Our data instead suggests an alternative pathway between ER and aggression: if people with MUD have lower sensitivity to positive emotions, and perceive subtle happy facial expressions as neutral, social situations might be experienced as hostile because of the lack of experienced positive emotional cues.

In addition, participants with MUD showed a decreased tendency to trust the investor in the trust game. Our study is the first to experimentally evaluate the propensity for people with MUD to engage in interpersonal trust. Our results align with previous results showing lower prosocial behaviour in cocaine users as well as the self‐report literature showing prosocial behaviour is impacted by addiction [47]. Interpersonal trust involves computations regarding both anticipated costs and benefits [19], and therefore, our findings suggest people with MUD may have difficulties managing risk and uncertainty in social scenarios involving decisions to act in self‐interest or in a prosocial manner. Evaluation of this complex social dilemma involves the recruitment of reward [20] plus central‐executive [21] neural networks, and methamphetamine use negatively impacts the function of these networks [22]. The tendency to extend interpersonal trust has important real‐world implications for social behaviour as illustrated by its role in predicting aggression among people with substance use disorders undergoing compulsory detoxification [24]. Of note, impairments in prosocial behaviour may be partially reversible if stimulant use is decreased or ceased as a longitudinal study suggested [48].

We also found that people with MUD applied greater punishment intensities to their confederates in the CRTT compared to controls. These data provide evidence that people with MUD show increased social aggression tendencies in experimental tasks. Experimental analogues that measure the behavioural tendency to punish another person are useful ways of observing whether people with MUD act aggressively in contexts without other social cues (e.g. body language, facial expressions) that might mediate such decisions. Only two prior studies used objective measures of aggression in MUD. Payer *et al*. [26] found that people with MUD have a similar predisposition to controls regarding proactive acts of aggression but became more reactive to the behaviour of others. Zacher *et al*. [14] explicitly tested for proactive/reactive tendencies by evaluating whether punishment intensities varied as a function of being punished on the prior trial, and in line with our results, found no evidence of trial‐by‐trial variation that would indicate reactive aggression. Instead, our results, like in Zacher *et al*. [14], showed that people with MUD applied greater levels of punishment regardless of condition, indicating a trait‐like tendency to express anger with punishment. Individual differences in aggression in the CRTT negatively correlated with trust game scores, indicating aggression and trust are social decisions that involve overlapping cognitive processes. CRTT scores also correlated positively with psychotic experiences and negatively with quality of life, highlighting the broader burden associated with aggression tendencies.

Contrasting initial assumptions, we found no evidence of empathy deficits in MUD. There was weak, marginal evidence that people with MUD showed less emotional empathy for positive stimuli compared to negative stimuli. This is consistent with our findings that ER is specifically degraded for happy emotions. This also coincides with prior evidence that cognitive and emotional empathy were reduced in people with MUD [7, 12, 13, 14]. Alternatively, a study that administered the MET found no deficits in cognitive, but in emotional empathy in recreational and dependent cocaine users [49]. Finally, we found that individual differences in emotional empathy for positive stimuli correlated negatively with anger expression, suggesting that stronger empathy for positive emotions can be a protective factor for anger expression. Taken together with the evidence reported above, this suggests that diminished ER and emotional empathy for positive emotions might facilitate greater anger expression in people with MUD.

There are limitations to note. Our ER assessment only included happiness and anger. Prior research has found evidence for degraded ER in other negative emotions (e.g. fear and sadness [5, 13]). Future research should assess emotion recognition sensitivity in these other emotions. Moreover, ER and trust were both shown to be degraded in our MUD sample, but we did not explicitly test how ER and trust deficits co‐occur to influence decision‐making, and future research may use modified tasks (e.g. emotion cueing in a trust game [50]). Moreover, missing data in the MET might have reduced our power to observed clearer effects like other studies [14]. Broader factors associated with MUD could be partly mediating our observations, including changes in attention, executive functions and memory [4]. Moreover, our sample was recruited via referrals from clinical services and is representative of people with MUD who are treatment‐seeking, but social cognitive profiles might differ for individuals not seeking treatment. Finally, our data are cross‐sectional, and longitudinal studies are necessary to understand how this social‐cognitive profile contributes to the development and maintenance of MUD.

## CONCLUSIONS

Our study provides a detailed characterisation of SC and decision‐making in MUD. This helps improve our understanding of how social functioning is impacted by the cognitive effects associated with MUD and suggest that interventions for MUD might benefit from targeting simpler SC and complex social decision‐making.

## AUTHOR CONTRIBUTIONS

**Justin Mahlberg:** Conceptualization (equal); data curation (equal); formal analysis (equal); visualization (equal); writing—original draft (equal); writing—review and editing (equal). **Lauren Hanegraaf:** Conceptualization (equal); writing—original draft (supporting); writing—review and editing (supporting). **Josua Zimmermann:** Conceptualization (equal); investigation (equal); methodology (equal); project administration (equal); writing—review and editing (equal). **David M. Cole:** Conceptualization (equal); investigation (equal); methodology (equal); project administration (equal); writing—review and editing (equal). **Boris B. Quednow:** Conceptualization (equal); formal analysis (equal); investigation (equal); methodology (equal); resources (equal); supervision (equal); writing—review and editing (equal). **Shalini Arunogiri:** Conceptualization (equal); investigation (equal); methodology (equal); project administration (equal); resources (supporting). **Antonio Verdejo‐Garcia:** Conceptualization (supporting); funding acquisition (supporting); methodology (supporting); project administration (supporting); resources (supporting); writing—review and editing (supporting).

## DECLARATION OF INTERESTS

None.

## Supporting information


**Figure S1.** Model diagnostics for the linear mixed effects model fit for the FMT task data.
**Figure S2.** Model diagnostics for the binomial mixed effects model fit for the MET cognitive task data.
**Figure S3.** Model diagnostics for the linear mixed effects model fit for the MET emotional data.
**Figure S4.** Model diagnostics for the linear mixed effects model fit for the trust game data.
**Figure S5.** Model diagnostics for the linear mixed effects model fit for the CRTT data.
**Table S1.** Linear model for Emotion recognition responses in the FMT. The data presented for the fixed effects represented the standardised coefficients (Beta), and their respective standardised error (SE) and 95% confidence interval (95CI). The random effects model adjusted the intercept to control for subject level variance, as well as adjusting the slope for variance due to changes in condition.
**Table S2.** Models assessing responses for the MET. A logit mixed effects model for predicting accuracy in the cognitive empathy test is presented on the left, and a linear mixed effects model for predicting responses on the emotional empathy test is presented on the right. For the logit model, the random effects model adjusted the intercept for subject level variance and variance for variance as function of the age of the character featured in the stimuli (adult or child). The emotional empathy model featured an identical random effects structure, along with the addition of adjusting the slope of the model for the valence of the stimuli. The data presented for the fixed effects represented the standardised coefficients (Beta), and their respective standardised error (SE) and 95% confidence interval (95CI).
**Table S3.** A linear mixed effects model assessing how the proportion of money that the participant trusts with the investor varies as a function of gender, education, and group over the 10 rounds. The data presented for the fixed effects represented the standardised coefficients (Beta), and their respective standardised error (SE) and 95% confidence interval (95CI). The random effects model adjusted the intercept to control for subject level variance.
**Table S4.** Shows the results of a mixed linear model assessing the effects of gender, education, group, whether punishment was experienced on the prior trial and the interaction between group and if they lost the previous trial experienced. The random effects model controlled for subject level variance in overall punishment intensities. The data presented for the fixed effects represented the standardised coefficients (Beta), and their respective standardised error (SE) and 95% confidence interval (95CI).
**Table S5.** Analysis of variance results for the emotion perception sensitivity for the FMT as a function of positive urine tests.
**Table S6.** Analysis of variance results for the accuracy on the cognitive empathy trials for the MET as a function of positive urine tests.
**Table S7.** Analysis of variance results for the responses on the emotional empathy trials for the MET as a function of positive urine tests.
**Table S8.** Analysis of variance results for the trust game responses as a function of positive urine tests.
**Table S9.** Analysis of variance results for the competitive reaction time task (CRTT) punishment intensity responses as a function of positive urine tests.

## Data Availability

Data available on request from the authors.
