# Supplementary material for: Social cognition and decision‐making in people with methamphetamine use disorder
Source: Addiction. 2025 Jun 16;120(10):2020–31. doi: 10.1111/add.70108 (PMC12426355; doi:10.1111/add.70108)
Supplement: Supplementary file 1 — Figure S1. Model diagnostics for the linear mixed effects model fit for the FMT task data. Figure S2. Model diagnostics for the binomial mixed effects model fit for the MET cognitive task data. Figure S3. Model diagnostics for the linear mixed effects model fit for the MET emotional data. Figure S4. Model diagnostics for the linear mixed effects model fit for the trust game data. Figure S5. Model diagnostics for the linear mixed effects model fit for the CRTT data. Table S1. Linear model for Emotion recognition responses in the FMT. The data presented for the fixed effects represented the standardised coefficients (Beta), and their respective standardised error (SE) and 95% confidence interval (95CI). The random effects model adjusted the intercept to control for subject level variance, as well as adjusting the slope for variance due to changes in condition. Table S2. Models assessing responses for the MET. A logit mixed effects model for predicting accuracy in the cognitive empathy test is presented on the left, and a linear mixed effects model for predicting responses on the emotional empathy test is presented on the right. For the logit model, the random effects model adjusted the intercept for subject level variance and variance for variance as function of the age of the character featured in the stimuli (adult or child). The emotional empathy model featured an identical random effects structure, along with the addition of adjusting the slope of the model for the valence of the stimuli. The data presented for the fixed effects represented the standardised coefficients (Beta), and their respective standardised error (SE) and 95% confidence interval (95CI). Table S3. A linear mixed effects model assessing how the proportion of money that the participant trusts with the investor varies as a function of gender, education, and group over the 10 rounds. The data presented for the fixed effects represented the standardised coefficients (Beta), and their respective stan [file ADD-120-2020-s001.docx]

**SUPPLEMENTARY INFORMATION FOR SOCIAL COGNITION AND DECISION-MAKING IN PEOPLE WITH METHAMPHETAMINE USE DISORDER**

**Authors:** Justin Mahlberg^1^, Lauren Hanegraaf^1^, Josua Zimmermann^2,3^, David M. Cole^2,4^, Boris B. Quednow^2,3^, Shalini Arunogiri^5,6^, Antonio Verdejo-Garcia^1^

1. *School of Psychological Sciences and Turner Institute for Brain and Mental Health, Monash University, Clayton, Victoria, Australia*
2. *Experimental Pharmacopsychology and Psychological Addiction Research, Department of Adult Psychiatry and Psychotherapy, University Hospital of Psychiatry Zurich, University of Zurich, Zurich, Switzerland*
3. *Neuroscience Center Zurich, University of Zurich and Swiss Federal Institute of Technology, Zurich, Switzerland*
4. *Translational Psychiatry Lab, University Psychiatric Clinics Basel, University of Basel, Basel, Switzerland*
5. *Monash Addiction Research Centre, Eastern Health Clinical School, Faculty of Medicine, Nursing and Health Sciences, Monash University, Melbourne, Victoria, Australia*
6. *Turning Point, Eastern Health, Melbourne, Victoria, Australia*

**SUPPLEMENTARY METHODS**

**Social Cognition Measures**

*Emotion Recognition: Face Morphing Task (FMT)*

In each trial of this task, a face was shown that could be morphed by the participant via horizontal mouse movements. A horizontal mouse movement toward the left morphed a face toward a happy facial expression, a horizontal mouse movement toward the right morphed a face to an angry facial expression, and the face remained as neutral expression when the mouse was in the centre. Mouse location data ranged from 0 (extreme left) to 100 (extreme right), and these values were used as an index of how sensitive an individual was to angry and happy facial expressions, while the neutral expression condition provided an index of the extent to which an individual showed distorted perceptions of neutral expressions (i.e. biased towards angry or happy). Once a participant decided upon the correct facial expression, they could click the mouse to confirm their response, though an interval of 5 seconds needed to pass before a confirmation response could be made, in order to prevent premature responding. There were 39 unique faces presented, which were created using 101 images for each face to enable participants to morph faces from angry through to a happy expression with a high resolution. There were three conditions within the task, and a participant was asked to use their mouse to: (1) find that the most neutral position (where there was no sign of either happiness of anger), (2) find a point where the face shows the first sign of anger, or (3) find a point where the face shows the first sign of happiness. Each condition included 13 trials, and the presentation order of the conditions was randomised for each participant. The faces presented for each condition was pseudorandomised, such that every participant received the same faces in the same order to ensure that an equal number of male and female faces were presented in each condition, and there were no more than two consecutive trials that showed a face of the same gender. For the first condition shown, there were two tutorial trials presented to ensure the participants understood what they were required to do before they preceded with the task. Generally, face emotion recognition tasks are reliable, with substantial agreement between participants when rating the emotion of faces (74.90% agreement; see 1). Moreover, evidence suggests that people can detect emotions at differing intensities, and people with MUD can also accurately discriminate anger and happiness when presented with emotions at lower intensities (2). Assessing perception for emotions at lower intensities is useful for revealing subtle differences, compared to healthy controls, in clinical groups including MUD (2, 3).

*Empathy: Multifaceted Empathy Test (MET)*

The MET is a computer-based task which uses photorealistic stimuli to assess cognitive and affective empathy. Participants were presented with 40 pictures of individuals of various ages, genders, and ethnicities in emotionally charged situations. Twenty images depicted positive/pleasant emotional valence and 20 depicted negative/unpleasant emotional valence (10 male/10 female, 8 adults/2 children for each valence). Images were rated separately for cognitive and emotional empathy and were presented in four blocks of 10 images, where the images were randomly selected. Cognitive empathy was assessed by asking participants to select the correct emotional label from four choices. Emotional empathy was assessed by asking participants to rate their level of empathy for the person in the image on a Likert scale of 1-9 (1 = not at all, 9 = very much). An accuracy measure of cognitive empathy was derived for each emotional valence condition by dividing the number of correct responses by the total number of trials. An average score for emotional empathy for positively and negatively valanced images was generated for each participant. The MET task has shown good validity, as the cognitive and emotional empathy tasks correlate more strongly with self report scales measuring the same subconstructs of empathy, in addition to good internal consistency (Cronbach's alpha = 0.71 for cognitive empathy and 0.91 for emotional empathy; 4). The MET has also been used to assess empathy in a wide range of clinical and non-groups, indicating that the task can be effectively administered in clinical samples, and is sensitive to selective impairments on specific emotion types depending on the underlying characteristics, neuropathology or psychopathology (5, 6).

**Social Decision-Making Measures**

*Interpersonal Trust: Trust game*

The trust game is a social interaction game in which participants play as investees (trustees) and are asked to collaborate with another person (the investor), which is – unbeknownst to the participant – simulated by the computer. In each round, the investor was given $20 and decided how much to keep for themselves and how much to invest in the stock market. The amount invested is multiplied by three, and given to the trustee as investment returns. The trustee then needed to decide at the end of each round how much of the market returns they would keep, and how much they would give to the investor. The main performance index for this task was the proportion of market returns invested by the participant, which is taken as a measure of interpersonal trust. Participants were provided with a cover story that advised the participant that they are playing against was an actual player, but for confidentiality they are not allowed to play in the same room, and instead there is another participant in another room performing the same task. Participants were instructed that after the task, they would be paid based on a scaled version of the total amount of money earned. The instructions that participants received were as follows:

*In this game you will play 10 rounds with the *same* partner. Your role for this game will be the "trustee" and your partner will be the "investor". A symbol will appear on the screen that will represent you for the game. In each round, the investor will decide how much money to keep and how much to invest. The amount invested will be multiplied by 3 and sent to you. You will decide how much of that money to keep and how much to send back to the investor. The totals for that round will be displayed on the screen. For each round, you will be given $20 to start. After 10 rounds, you will be paid based on a scaled version of the total amount of money you earn in the game.*

The investors responses (investment values) were simulated using a k-nearest neighbour algorithm to draw responses from a training dataset with pairs of humans playing the trust game. For the first round, the algorithm randomly chooses an investment value between $0 and $20. On subsequent rounds (rounds 2-10), the algorithm draws a response by observing the recent history of its own responses (up to 6 prior responses), and compares this to the 6 prior responses for human investors in the training dataset. Euclidean distance was calculated to find six different sets of responses from the training dataset that closely match the response patterns of the algorithm in the current game:

*Distance = √[(x₁ − y₁)² + (x₂ − y₂)² + ... + (x₆ − y₆)²]*

In this formula, x₁ to x₆ are the bot’s investment values in the most recent rounds (up to 6), and y₁ to y₆ are the corresponding investments made by a human investor in the training dataset. This distance score tells the algorithm how closely the bot’s current behavior matches each human example. The algorithm identifies the six most similar sequences (i.e., those with the smallest distances), and samples its next investment value from the real choices that followed each of those sequences. This allows the bot to mimic how real investors behaved in similar situations, producing human-like investment patterns throughout the game.

Behaviour in the trust game is a valid measure of interpersonal trust as it is associated with self-report measures of social trust (7), correlated with related constructs such as personality traits such as honesty and humility (8), and negatively correlated with beliefs that would be expected to reduce trust behaviour (9). While correlated with related constructs, evidence suggests that this paradigm does measure trust behaviours that are distinguishable from related constructs like altruism (10).

*Aggression: The Competitive Reaction Time Task (CRRT)*

Participants were told that they were competing against an opponent and that they had to try to outperform this opponent by clicking the mouse when the screen colour changed to green. The CRRT was 25 trials in length. Participants were provided the following instructions: “*If you win your opponent will be blasted with a sound, if you lose you will have a sound blasted at you. Before each trial you can set the duration and the volume of the sound for your opponent should you win.”.* The researcher administering the experiment emphasised that this opponent was different to the other opponent the participant versed in the other tasks, to reduce carryover beliefs about the opponent from the other tasks. Participants could choose to blast their opponent with a noise set between 8 levels of volume, where level 1 was equivalent to 55 dCA and level 8 was equivalent to 95 dCA. The performance of the opponent was simulated so that the level of punishment the opponent assigned for the participant to receive was pseudo-randomised. The outcome (win/loss) of each trial depended on the participant’s reaction time and the outcome of the previous trials, insofar as a participant won against an opponent only if (1) their response was faster than their median reaction time in prior trials and (2) they had not won >50% of prior trials. The CRTT is a valid measures of aggression, as behaviour captured in this task correlate with self-report aggression (11), and traits linked to aggressive behaviour (12). P-curve analysis shows these lab-based measures generate reliable effects of substantial evidentiary value (13). Moreover, the CRTT has been used previously to explore aggression behaviour in people with MUD (14, 15).

**SUPPLEMENTARY RESULTS**

**Model fitting and diagnostic assessment**

Cognitive task data were fit with multilevel models using lmerTest, and Satterthwaite estimation was used to produce degrees of freedom and significance tests. Random effects models were selected by fitting the maximal unconditional random effects structures to account for known sources of variation from the experimental design. In cases where maximal random effect structures were ill fitting (i.e. the model did not converge), random effect structures were iteratively simplified by removing variables that explained near zero variance or removing slope random effects. Model diagnostics were produced using the *performance* package to ensure the models conformed to their respective assumptions. We describe here the details of the model fitting and diagnostics assessment process for each model reported in the manuscript.

**Facial morphing task**

Emotion recognition sensitivity was assessed by analysing facial morphing responses. The trial conditions were either asking people to identify the first sign of a happy face, an angry face, or to identify a neutral face (Condition). We fit the data with a linear mixed effects model, where the random effects structure included a random intercept for subject to account for individual differences in responses, and a random intercept for Condition to account for individual differences in the extent to which the condition effects a participants responses on the task. The fixed effects included gender and education as covariates, and tested for a main effect of task condition (happy, angry, neutral), group (CTRL, MUD) and their interaction. Equation 1 below corresponds to the final model fit for the cognitive empathy data:

$$\left( 1 \right) Emotion recognition=Gender+Education +Condition *Group+\left( Condition \right| subject)$$

The final model fit the data appropriately and largely conformed to the respective assumptions for a logit model. Figure S1 shows model diagnostics for the FMT emotion recognition sensitivity data. There was some indication that responses deviated from a normal distribution around the extreme points of the response scale, indicating some potential censoring. However, overall the model fit the data appropriately and conformed to the assumptions of a mixed linear effects model. We report the full model fit results in Table S1.

**Multifaceted Empathy task – cognitive empathy**

Cognitive empathy was assessed by analysing whether participants correctly identified the emotion in the stimuli shown in each trial. The stimuli were either positively or negatively valanced (Stimuli Valance), and some stimuli featured children whereas others featured adults (Stimuli Age). We fit the data with a logit mixed effects model, where the random effects structure included a random intercept for subject to account for individual differences in responses, and a random intercept for stimuli age to account for potential stimuli-driven variance in cognitive empathy scores due to the age of the person featured in the stimuli. The fixed effects included gender and education as covariates, and tested for a main effect of stimuli valence (positive, negative), group (CTRL, MUD) and their interaction. Equation 2 below corresponds to the final model fit for the cognitive empathy data:

$$\left( 2 \right) Cognitive Empathy=Gender+Education +Stimuli Valence *Group+\left( 1 \right| subject)+(1 | Stimuli Age)$$

To achieve the final model, we removed stimuli valence as a random effect, as including this as either a random slope or intercept resulted in a singular model fit, as valence explained near-zero variance in cognitive empathy scores. The final model fit the data appropriately and largely conformed to the respective assumptions for a logit model. Figure S2 shows the model diagnostics for the MET cognitive empathy responses. We report the full model fit results in Table S2 (Cognitive Empathy).

**Multifaceted Empathy task – emotional empathy**

Emotional empathy was assessed by analysing the rating that participants provided for their experience of empathy when viewing the stimuli in each trial. The stimuli were either positively or negatively valanced (Stimuli Valance), and some stimuli featured children whereas others featured adults (Stimuli Age). The distribution of all emotional empathy scores deviated from a normal distribution and we summarised the data to the level of average empathy responses for each condition in each participant, which improved adherence to a normal distribution and subsequently improved model fit. We fit the data with a linear mixed effects model, where the random effect structure included a random intercept for subject to account for individual differences in responses, and a random slope for stimuli valence to account for individual differences in the extent to which empathy scores were impacted by valence within each participant. We also included a random intercept for stimuli age to account for potential stimuli-driven variance in emotional empathy scores due to the age of the person featured in the stimuli. The fixed effects included gender and education as covariates, and tested for a main effect of stimuli valence (positive, negative), group (CTRL, MUD) and their interaction. Equation 3 below corresponds to the final model fit for the emotional empathy data:

$$\left( 3 \right) Emotional Empathy=Gender+Education +Stimuli Valence *Group+\left( Valence \right| subject)+(1 | Stimuli Age)$$

The final model fit the data appropriately and largely conformed to the respective assumptions for a logit model. Figure S3 shows the model diagnostics for the MET emotional empathy responses. We report the full model fit results in Table S2 (Emotional Empathy).

**Trust Game**

Interpersonal trust was assessed by analysing the proportion of market returns that a participant received on each round that they returned to the investor. We fit the data with a linear mixed effects model, where the random effect structure included a random intercept for subject to account for individual differences in responses. We began fitting the model with a random effect for round to account for potential individual differences in the extent to which prosocial behaviour changed for each person as the rounds progressed. However, round explained near zero variance and thus was removed from the final model. The fixed effects included gender and education as covariates, and tested for a main effect of group (CTRL, MUD) and their interaction. Equation 4 below corresponds to the final model fit for the emotional empathy data:

$$\left( 4 \right) \% market returns=Gender+Education +Group+\left( 1 \right| subject)$$

The final model fit the data appropriately and largely conformed to the respective assumptions for a logit model. Figure S4 shows the model diagnostics for the trust game. We report the full model fit results in Table S2 (Emotional Empathy).

***Competitive reaction time task***

Aggressing was assessed by analysing the punishment intensity settings that participants provided for each trial. The punishment intensity settings were selected after losing the trial before and receiving a punishment of an intensity selected by their opponent (provoked) or after winning the trial before, and therefore did not receive a punishment (unprovoked). The distribution of all punishment intensity settings deviated from a normal distribution and we summarised the data to the level of average punishment intensity responses for each condition (provoked or unprovoked) in each participant, which improved adherence to a normal distribution and subsequently improved model fit. We fit the data with a linear mixed effects model, where the random effect structure included a random intercept for subject to account for individual differences in responses. The fixed effects included gender and education as covariates, and tested for a main effect of Condition (provoked, unprovoked), group (CTRL, MUD) and their interaction. Equation 3 below corresponds to the final model fit for the emotional empathy data:

$$\left( 3 \right) Aggression=Gender+Education +Condition *Group+\left( 1 \right| subject)$$

The final model fit the data appropriately and largely conformed to the respective assumptions for a linear mixed effects model. Figure S5 shows the model diagnostics for the MET emotional empathy responses. We report the full model fit results in Table S4.

**SENSITIVITY ANALYSES**

**MUD group fatigue**

We utilised a measure of fatigue at the beginning (*M* = 42.72, *SD* = 13.91) and end of the assessment battery (*M* = 43.35, *SD* = 14.70) and found no evidence that levels of fatigue changed during the assessment battery, *t*(50) = .92 , *p* = 0.36, *95CI* = [-1.43, 3.86].

**MET manipulation check**

In terms of the experimental manipulation, the CTRL group showed happy responses that were significantly lower, *t*(92) = 6.84, *p <* 0*.*0001, and angry responses that were significantly higher than neutral responses, *t*(92) = 7.50, *p <* 0*.*0001. MUD participants also showed happy responses that were lower, *t*(92) = 7.46, *p <* 0*.*0001, and angry responses that were higher than neutral responses, *t*(92) = 11.97, *p <* 0*.*0001.

**Does recent other drug use in people with MUD impact task effects?**

One might question whether recent other drug use might have impacted the group effects observed. To examine this question, we probed whether the presence or absence of drugs other than methamphetamines in the urine tests impacted responses of people with MUD in the social cognition and decision-making tasks (results reported in Tables S5-S9). Emotion perception was consistent across individuals with MUD regardless of their recent drug use. Cognitive empathy was slightly higher overall for people with recent opiate use, but recent opiate use did not interact with the effect of valence. There was no evidence that emotional empathy was impacted by recent use of other drugs. There was no evidence that recent other drug use impacted trust game behaviour. Recent THC use predicted lower punishment levels, indicating that recent THC use reduced aggressive tendencies in people with MUD. The presence of other drugs did not impact aggressive behaviour in the CRTT.

**Extended Covariate models – controlling for QoL, depression, anger expression, and psychotic experiences**

To examine whether group differences in psychosocial outcomes, including quality of life, depression, anger expression, and psychotic experiences could explain the group effects reported in the manuscript, we included these variables as covariates in our models and report the results below. The results reported below are consistent with the pattern in results reported in the main manuscript.

*Facial Morphing Task*

After controlling for gender, education, quality of life, anger expression, depression, and psychotic experiences, the emerging pattern was consistent with what we reported in the manuscript. There was no evidence for main effects of gender, education, quality of life, anger expression, depression, and psychotic experiences (all *F* < 2.39, all *p* > 0.12). There was a main effect of Condition, *F*(2,94) = 79.69, *p <* 0.001, and group, *F*(1, 112.21) = 6.54, *p* = 0.012, and an interaction between Condition and group, *F*(2, 94) = 5.23, *p* = 0.007. MUD showed a significant bias toward perceiving neutral faces as happier compared to the CTRL group, *t*(112) = 2.14, *p* = 0.01, and showed lower sensitivity to happy expressions, *t*(127) = 2.45, *p* = 0.02, whereas groups were similar in their sensitivity to angry expressions, *t*(127) = 1.11, *p* = 0.27.

*Multifaceted Empathy task (cognitive empathy)*

After controlling for gender, education, quality of life, anger expression, depression, and psychotic experiences, the emerging pattern was consistent with what we reported in the manuscript. There was a main effect of valence, *𝜒^2^*(1) *= 41.55, p <* 0.001. All other main effects and interactions were non-significant (all 𝜒^2^ < 2.03, p > 0.15).

*Multifaceted Empathy task (emotional empathy)*

After controlling for gender, education, quality of life, anger expression, depression, and psychotic experiences, the emerging pattern was consistent with what we reported in the manuscript. Valence, *F*(1,84) = 0.46, *p* = 0.50, or group, *F*(1, 99.44) = 0.46, *p* = 0.10 or their interaction, *F*(1, 84) = 3.51, *p* = 0.07, impacted emotional empathy responses. The marginal interaction was driven by the MUD group, which was marginally less emotionally empathetic to positive stimuli compared to negative stimuli, *t*(84) = 1.90, *p* = 0.06, whereas the CRTL group showed no differences across stimulus valence, *t*(84) = .68, *p* = 0.50.

*Trust game*

After controlling for gender, education, quality of life, anger expression, depression, and psychotic experiences, the emerging pattern was consistent with what we reported in the manuscript. There was a significant group difference, such that MUD entrusted a lower proportion of the market returns to the investor compared to the CTRL group, *F*(1, 85.82) = 7.04, *p* = 0.001. There was no evidence that the covariates impacted responses (all *F* < 2.26, all *p* > 0.13).

*Competitive reaction time task (aggression)*

After controlling for gender, education, quality of life, anger expression, depression, and psychotic experiences, the emerging pattern was consistent with what we reported in the manuscript. The MUD group selected more intense punishments for their opponent compared to the CTRL group, *F*(1, 93.00) = 7.45, *p* = 0.008. There was no evidence of retaliation, insofar as punishment intensity was similar whether or not punishments were experienced on the prior trial, *F*(1, 93) = 2.17, *p* = 0.14, or for an interaction with group, *F*(1, 93) = 0.22, *p* = 0.64. There was no evidence that the other covariates impacted responses (all *F* < 2.7, all *p* > 0.10).

**Is the presence of current drug-induced psychosis driving group effects?**

To examine whether the sub group of participants with MUD who also had current drug-induced psychosis symptoms were driving the group effects reported in the manuscript, we examined the presence or absence of current drug-induced psychosis as a grouping variable for the social-cognition tasks, and report below that there was no evidence for the presence of drug-induced psychosis symptoms impacting task performance, suggesting that current drug-induced psychosis is unlikely to explain the patterns reported in the manuscript.

*Facial Morphing Task*

There was no evidence that current psychosis impacted the results, as there was no effect of psychosis on performance overall, *F*(1, 44.30) = 0.04, *p* = 0.84, while there was a main effect of the task conditions, *F*(1, 44.00) = 43.27, *p <* 0.001, and no interaction, *F*(2, 44.00) = 0.91, *p* = 0.41. The was no effect of gender, *F*(1, 43) = 1.66,  *p* = 0.20, or education, *F*(1, 43) = 1.08, *p* = 0.31.

*Multifaceted Empathy task (cognitive empathy)*

There was no evidence that current psychosis impacted the results, as there was no effect of psychosis status on performance, 𝜒^2^ = 2.65, *p* = 0.10. While there was a main effect of valence, 𝜒^2^= 12.35, *p <* 0.001, there was no evidence that this interacted with psychosis status, 𝜒^2^ = 0.85, *p* = 0.36. There was no impact of gender, 𝜒^2^ = 0.04, *p* = 0.85, but education did impact performance, 𝜒^2^= 7.63, *p* = 0.005.

*Multifaceted Empathy task (emotional empathy)*

There was no evidence that current psychosis impacted the results, as there was no effect of psychosis status on performance, *F*(1, 34) = 0.85, *p* = 0.37. There was no evidence for an effect of valence, *F*(1, 36) = 1.33, *p* = 0.25, nor the interaction between valence and psychosis status, *F*(1, 36) = 0.77, *p* = 0.39. The was no effect of gender, *F*(1, 34) = 0.89,  *p* = 0.35, or education, *F*(1, 34) = 0.01, *p* = 0.91.

*Trust game*

There was no evidence that current psychosis impacted the results, as there was no effect of psychosis status on performance, *F*(1, 42.06) = 0.01, *p* = 0.75. The was no effect of gender, *F*(1, 42.26) = 0.0002,  *p* = 0.99, or education, *F*(1, 41.87) = 1.70, *p* = 0.20.

*Competitive reaction time task (aggression)*

There was no evidence that current psychosis impacted the results, as there was no effect of psychosis status on performance, *F*(1, 46.59) = 0.08, *p* = 0.78. There was also no evidence that current psychosis impacted provoked punishment, *F*(1, 45) = 0.91, *p* = 0.34. There was no evidence for a pain effect of provoked punishment, *F*(1, 45) = 0.03, *p* = 0.87, nor was there evidence that gender, *F*(1, 43) = 0.70,  *p* = 0.35, or education, *F*(1, 43) = 1.48, *p* = 0.91, impacted the results.

**SUPPLEMENTARY FIGURES**


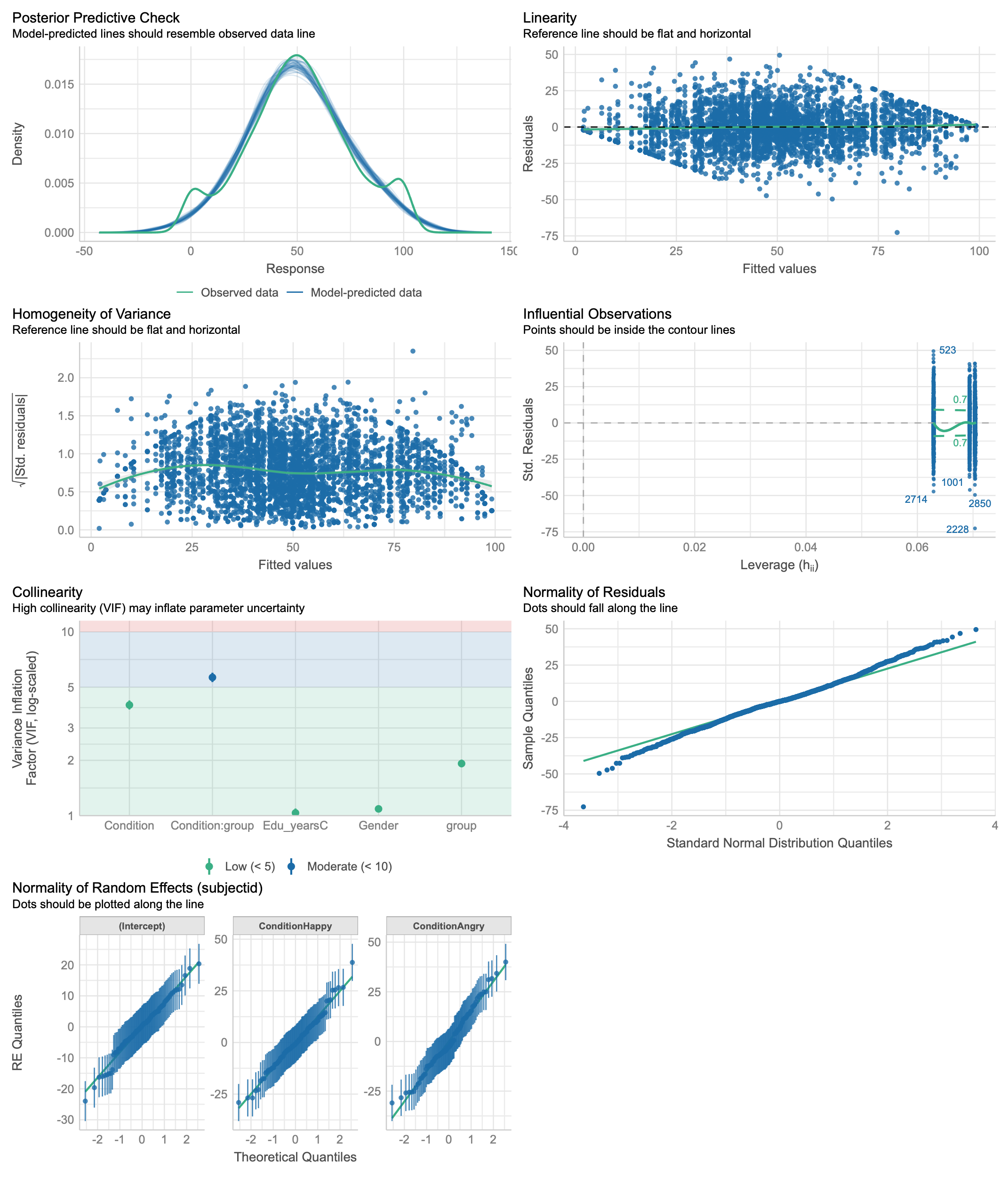


Figure S1. Model diagnostics for the linear mixed effects model fit for the FMT task data.


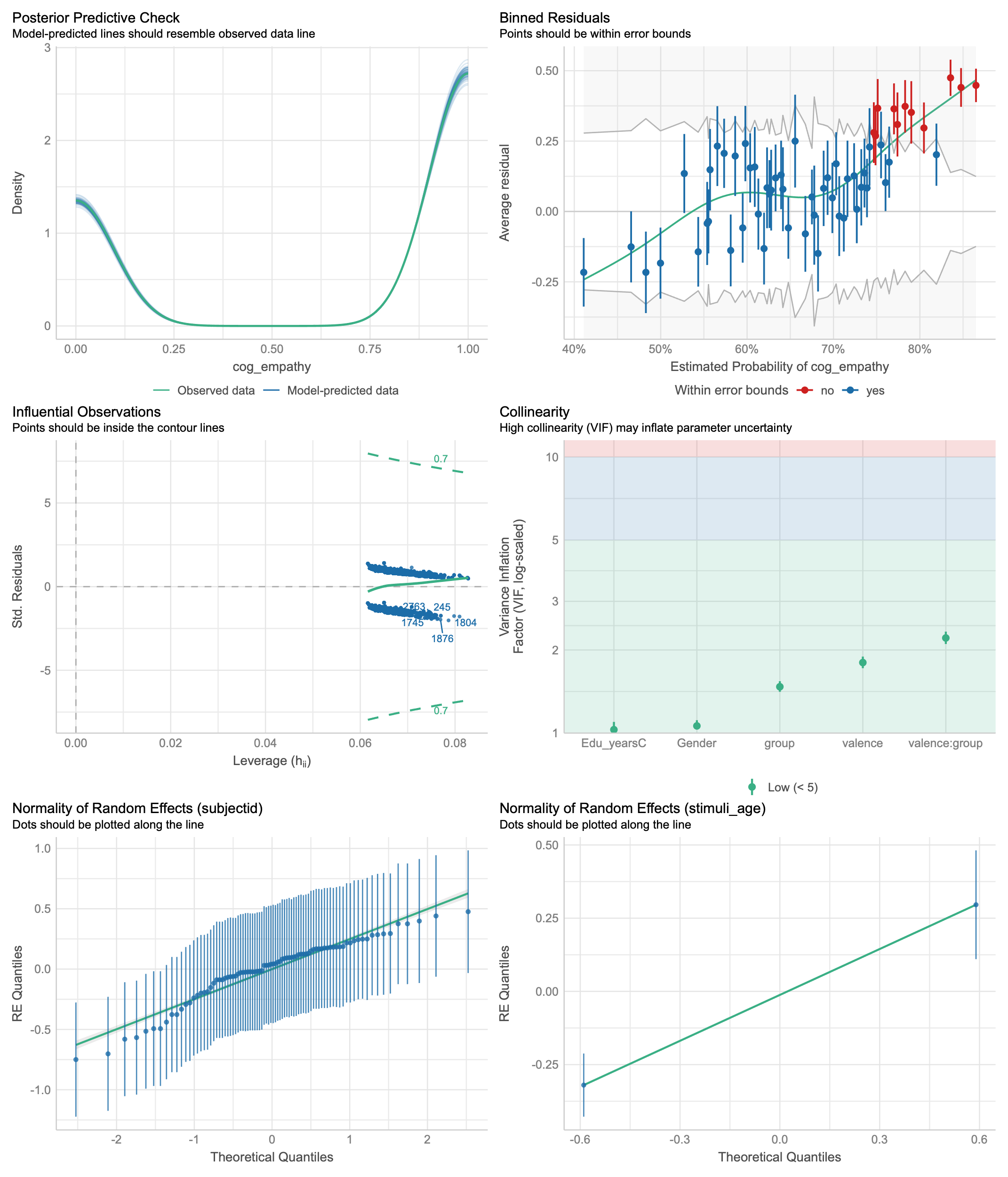


Figure S2. Model diagnostics for the binomial mixed effects model fit for the MET cognitive task data.


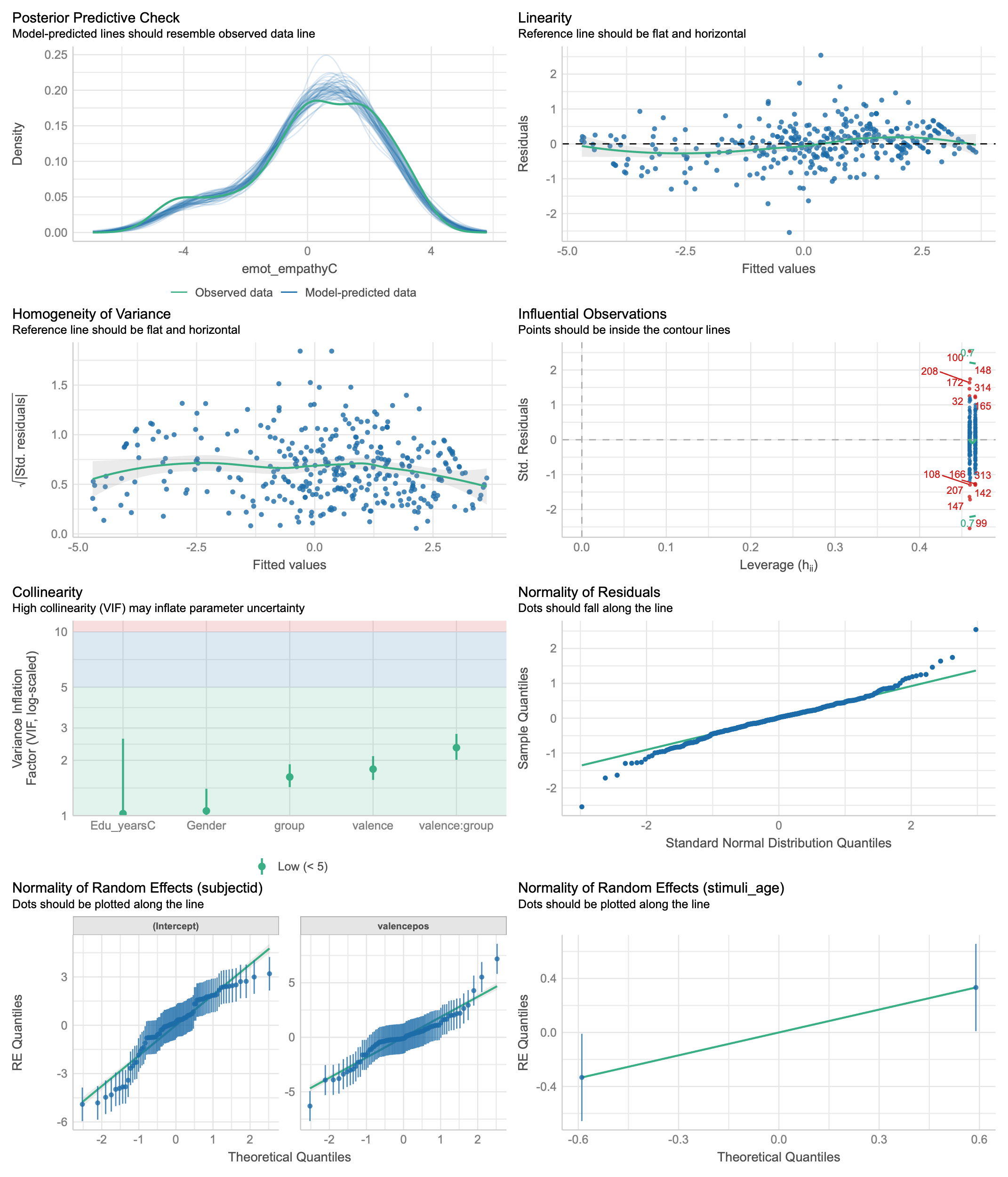


Figure S3. Model diagnostics for the linear mixed effects model fit for the MET emotional data.


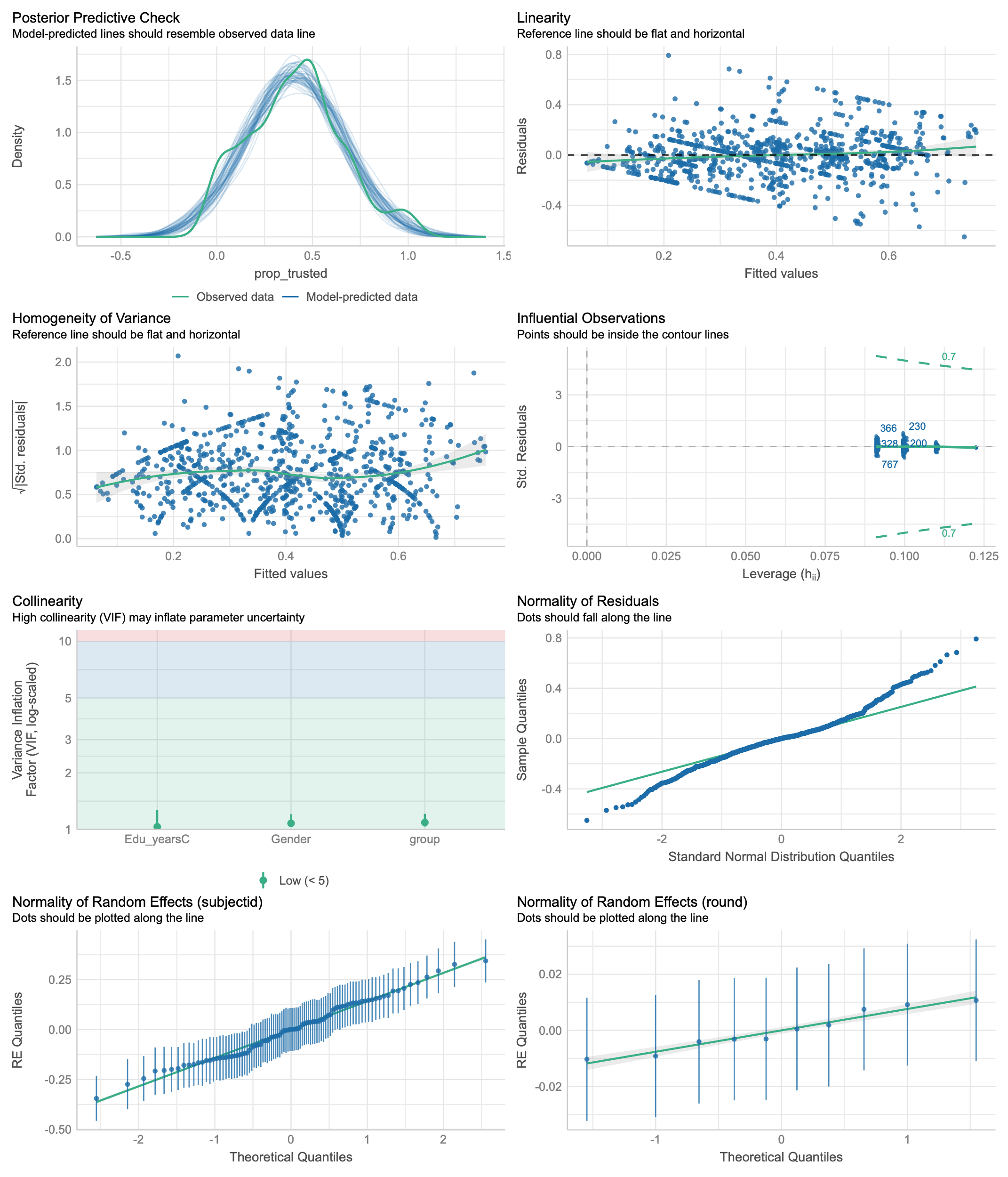


Figure S4. Model diagnostics for the linear mixed effects model fit for the trust game data.


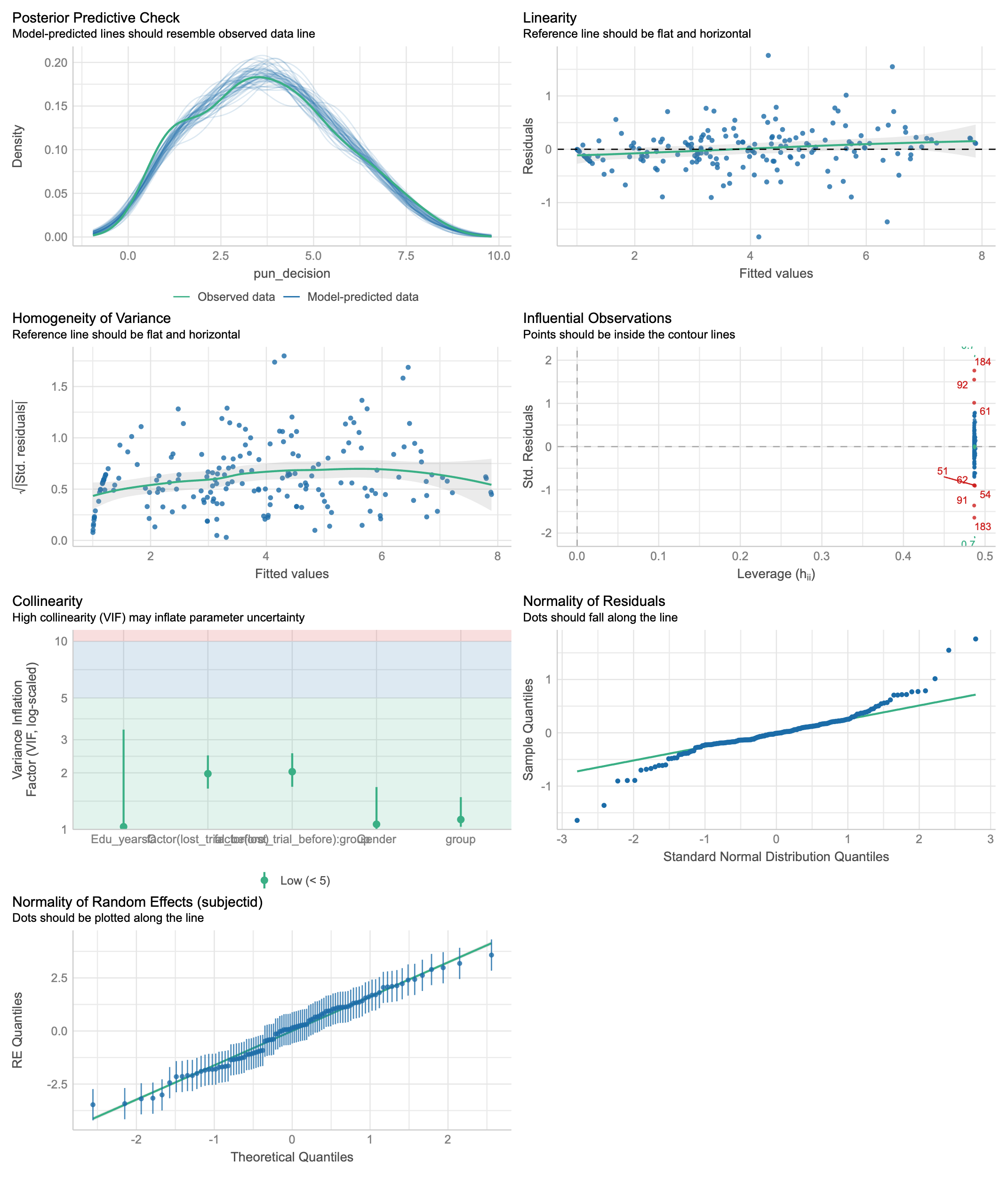


Figure S5. Model diagnostics for the linear mixed effects model fit for the CRTT data.

**SUPPLEMENTARY TABLES**

Table S1.

Linear model for Emotion recognition responses in the FMT. The data presented for the fixed effects represented the standardised coefficients (Beta), and their respective standardised error (SE) and 95% confidence interval (95CI). The random effects model adjusted the intercept to control for subject level variance, as well as adjusting the slope for variance due to changes in condition.

|  | **Emotion recognition** | | |
| --- | --- | --- | --- |
| *Predictors* | *Beta* | *SE* | *95CI* |
| (Intercept) | -0.03 | 0.07 | -0.17 – 0.10 |
| Gender [Male] | 0.03 | 0.07 | -0.10 – 0.16 |
| Education (years) | -0.01 | 0.03 | -0.07 – 0.06 |
| Condition [Happy] | -0.59 | 0.09 | -0.76 – -0.42 |
| Condition [Angry] | 0.76 | 0.10 | 0.56 – 0.96 |
| Group [MUD] | -0.21 | 0.08 | -0.38 – -0.04 |
| Condition [Happy] × Group [MUD] | -0.05 | 0.12 | -0.29 – 0.19 |
| Condition [Angry] × Group [MUD] | 0.42 | 0.14 | 0.14 – 0.71 |
| **Random Effects** | | | |
| σ^2^ | 173.09 | | |
| τ_00_ _subjectid_ | 80.66 | | |
| τ_11_ _subjectid.ConditionHappy_ | 179.67 | | |
| τ_11_ _subjectid.ConditionAngry_ | 255.50 | | |
| ρ_01_ | -0.22 | | |
|  | -0.45 | | |
| ICC | 0.49 | | |
| N _subjectid_ | 94 | | |
| Observations | 3666 | | |
| Marginal R^2^ / Conditional R^2^ | 0.434 / 0.710 | | |

Table S2.

Models assessing responses for the MET. A logit mixed effects model for predicting accuracy in the cognitive empathy test is presented on the left, and a linear mixed effects model for predicting responses on the emotional empathy test is presented on the right. For the logit model, the random effects model adjusted the intercept for subject level variance and variance for variance as function of the age of the character featured in the stimuli (adult or child). The emotional empathy model featured an identical random effects structure, along with the addition of adjusting the slope of the model for the valence of the stimuli. The data presented for the fixed effects represented the standardised coefficients (Beta), and their respective standardised error (SE) and 95% confidence interval (95CI).

|  | **Cognitive Empathy** | | | **Emotional Empathy** | | |
| --- | --- | --- | --- | --- | --- | --- |
| *Predictors* | *Beta* | *SE* | *95CI* | *Beta* | *SE* | *95CI* |
| (Intercept) | 1.84 | 0.47 | 1.12 – 3.02 | -0.14 | 0.24 | -0.76 – 0.49 |
| Gender [Male] | 0.99 | 0.11 | 0.79 – 1.24 | 0.11 | 0.19 | -0.26 – 0.48 |
| Education (years) | 1.08 | 0.06 | 0.97 – 1.20 | -0.05 | 0.09 | -0.22 – 0.13 |
| Valence [Positive] | 1.91 | 0.19 | 1.57 – 2.33 | 0.10 | 0.15 | -0.20 – 0.40 |
| Group [MUD] | 1.07 | 0.14 | 0.83 – 1.39 | 0.25 | 0.22 | -0.19 – 0.69 |
| Valence [Positive] × group [MUD] | 0.88 | 0.13 | 0.65 – 1.18 | -0.43 | 0.23 | -0.88 – 0.03 |
| **Random Effects** | | | | | | |
| σ^2^ | 3.29 | | | 0.56 | | |
| τ_00_ | 0.13 _subjectid_ | | | 4.16 _subjectid_ | | |
|  | 0.10 _stimuli_age_ | | | 0.23 _stimuli_age_ | | |
| τ_11_ |  | | | 4.28 _subjectid.valencepos_ | | |
| ρ_01_ |  | | | -0.59 _subjectid_ | | |
| ICC | 0.07 | | | 0.88 | | |
| N | 86 _subjectid_ | | | 86 _subjectid_ | | |
|  | 2 _stimuli_age_ | | | 2 _stimuli_age_ | | |
| Observations | 3440 | | | 344 | | |
| Marginal R^2^ / Conditional R^2^ | 0.026 / 0.090 | | | 0.018 / 0.880 | | |

Table S3.

A linear mixed effects model assessing how the proportion of money that the participant trusts with the investor varies as a function of gender, education, and group over the 10 rounds. The data presented for the fixed effects represented the standardised coefficients (Beta), and their respective standardised error (SE) and 95% confidence interval (95CI). The random effects model adjusted the intercept to control for subject level variance.

|  | **Proportion of Market returns** | | |
| --- | --- | --- | --- |
| *Predictors* | *Beta* | *SE* | *95CI* |
| (Intercept) | 0.16 | 0.13 | -0.10 – 0.42 |
| Gender [Male] | 0.07 | 0.15 | -0.23 – 0.38 |
| Education (years) | 0.06 | 0.07 | -0.08 – 0.20 |
| Group [MUD] | -0.46 | 0.15 | -0.75 – -0.17 |
| Random Effects | | | |
| σ^2^ | 0.03 | | |
| τ_00_ _subjectid_ | 0.02 | | |
| ICC | 0.41 | | |
| N _subjectid_ | 94 | | |
| Observations | 889 | | |
| Marginal R^2^ / Conditional R^2^ | 0.051 / 0.445 | | |

Table S4.

Shows the results of a mixed linear model assessing the effects of gender, education, group, whether punishment was experienced on the prior trial and the interaction between group and if they lost the previous trial experienced. The random effects model controlled for subject level variance in overall punishment intensities. The data presented for the fixed effects represented the standardised coefficients (Beta), and their respective standardised error (SE) and 95% confidence interval (95CI).

|  | **Punishment intensity settings** | | |
| --- | --- | --- | --- |
| *Predictors* | *Beta* | *SE* | *95CI* |
| (Intercept) | -0.44 | 0.17 | -0.78 – -0.09 |
| Gender [Male] | 0.03 | 0.20 | -0.37 – 0.43 |
| Edu years | -0.09 | 0.10 | -0.27 – 0.10 |
| Condition [Lost previous trial] | 0.09 | 0.06 | -0.03 – 0.20 |
| Group [MUD] | 0.77 | 0.20 | 0.38 – 1.17 |
| Condition [Lost previous trial] × Group [MUD] | -0.04 | 0.08 | -0.20 – 0.13 |
| **Random Effects** | | | |
| σ^2^ | 0.30 | | |
| τ_00_ _subjectid_ | 2.86 | | |
| ICC | 0.91 | | |
| N _subjectid_ | 95 | | |
| Observations | 190 | | |
| Marginal R^2^ / Conditional R^2^ | 0.160 / 0.921 | | |

Table S5.

Analysis of variance results for the emotion perception sensitivity for the FMT as a function of positive urine tests.

| **FMT** | **Gender** | **Education** | **Condition** | **Positive test** | **Condition*Positive test** |
| --- | --- | --- | --- | --- | --- |
| Benzodiazepines | 2.22 | 1.03 | 49.21*** | 0.36 | 1.57 |
| THC | 1.95 | 1.42 | 45.02*** | 0.1194865 | 0.68 |
| Opiates | 1.89 | 0.80 | 50.24*** | 0.29 | 0.58 |
| Amphetamines | 2.39 | 1.29 | 15.40*** | 0.05 | 0.48 |
| MDMA | 3.41 | 0.62 | 48.20*** | 0.26 | 2.74 |
| Note: Reports F statistics for analysis of variance tests. We did not analyse positive cases for cocaine as there were only 2 positive cases. ***p < 0.001; ** P < 0.01, * P < 0.05. | | | | | |

Table S6.

Analysis of variance results for the accuracy on the cognitive empathy trials for the MET as a function of positive urine tests.

| **MET Cognitive empathy** | **Gender** | **Education** | **Valence** | **Positive test** | **Valence*Positive test** |
| --- | --- | --- | --- | --- | --- |
| Benzodiazepines | 0.26 | 6.97** | 9.50** | 0.52 | 0.36 |
| THC | 0.20 | 6.92** | 15.83*** | 0.09 | 0.34 |
| Opiates | 0.15 | 6.18* | 11.08*** | 5.21* | 0.32 |
| Amphetamines | 0.12 | 7.07** | 2.45 | 0.004 | 0.40 |
| MDMA | 0.08 | 7.06** | 13.94*** | 0.06 | 0.02 |
| Note: Reports 𝜒^2^ statistics for analysis of variance tests. We did not analyse positive cases for cocaine as there were only 2 positive cases. ***p < 0.001; ** P < 0.01, * P < 0.05. | | | | | |

Table S7.

Analysis of variance results for the responses on the emotional empathy trials for the MET as a function of positive urine tests.

| **MET Emotional empathy** | **Gender** | **Education** | **Valence** | **Positive test** | **Valence*Positive test** |
| --- | --- | --- | --- | --- | --- |
| Benzodiazepines | 1.21 | 0.01 | 0.62 | 1.39 | 1.54 |
| THC | 1.00 | 0.03 | 1.18 | 0.23 | 1.22 |
| Opiates | 1.86 | 0.19 | 2.42 | 0.09 | 0.03 |
| Amphetamines | 0.59 | 0.01 | 0.77 | 1.81 | 0.00 |
| MDMA | 0.43 | 0.20 | 5.89* | 1.09 | 0.33 |
| Note: Reports F statistics for analysis of variance tests. We did not analyse positive cases for cocaine as there were only 2 positive cases. ***p < 0.001; ** P < 0.01, * P < 0.05. | | | | | |

Table S8.

Analysis of variance results for the trust game responses as a function of positive urine tests.

| **Trust game** | **Gender** | **Education** | **Positive test** |
| --- | --- | --- | --- |
| Benzodiazapine | 0.14 | 2.17 | 2.17 |
| THC | 0.005 | 1.75 | 0.35 |
| Opiates | 0.02 | 1.71 | 0.15 |
| Amphetamines | 0.006 | 2.29 | 0.22 |
| MDMA | 0.13 | 2.01 | 0.06 |
| Note: Reports F statistics for analysis of variance tests. We did not analyse positive cases for cocaine as there were only 2 positive cases. ***p < 0.001; ** P < 0.01, * P < 0.05. | | | |

Table S9.

Analysis of variance results for the competitive reaction time task (CRTT) punishment intensity responses as a function of positive urine tests.

| **CRTT** | **Gender** | **Education** | **Lost prior trial** | **Positive test** | **Valence*Positive test** |
| --- | --- | --- | --- | --- | --- |
| Benzodiazepines | .36 | 0.74 | 0.93 | 3.97 | 0.20 |
| THC | 0.50 | 2.92 | 0.83 | 5.35* | 0.25 |
| Opiates | 0.41 | 0.75 | 2.20 | 2.03 | 3.98 |
| Amphetamines | 0.79 | 1.38 | 0.89 | 0.28 | 0.39 |
| MDMA | 0.33 | 0.97 | 0.43 | 0.30 | 0.01 |
| Note: Reports F statistics for analysis of variance tests. We did not analyse positive cases for cocaine as there were only 2 positive cases. ***p < 0.001; ** P < 0.01, * P < 0.05. | | | | | |

REFERENCES

1. Gourlay C, Pascal C, Pier-Olivier C, Camille DA, and Scherzer PB. Psychometric assessment of social cognitive tasks. Applied Neuropsychology: Adult. 2022;29(4):731-49.

2. Uhlmann A, Ipser JC, Wilson D, Stein DJ. Social cognition and aggression in methamphetamine dependence with and without a history of psychosis. Metab Brain Dis. 2018;33(2):559-68.

3. Bento de Souza IBM, Barbosa FF, Lacerda AM, dos Santos NA, Torro-Alves N. Evaluation of facial expressions in women with major depression: Is there a negative bias? Psychol Neurosci. 2014;7(4):513-9.

4. Dziobek I, Rogers K, Fleck S, Bahnemann M, Heekeren HR, Wolf OT, et al. Dissociation of Cognitive and Emotional Empathy in Adults with Asperger Syndrome Using the Multifaceted Empathy Test (MET). J Autism Dev Disord. 2008;38(3):464-73.

5. Montagne B, Kessels RPC, De Haan EHF, Perrett DI. The Emotion Recognition Task: A Paradigm to Measure the Perception of Facial Emotional Expressions at Different Intensities. Percept Mot Skills. 2007;104(2):589-98.

6. Fernandes Vieira de Lima F, Rossi G, dos Santos RG, de Lima Osório F. Multifaceted Empathy Test (MET): Validity evidence for the Brazilian population concerning the computer-based (face-to-face) and online versions. PLoS One. 2023;18(7):e0284524.

7. Safra L, Lettinga N, Jacquet PO, Chevallier C. Variability in repeated economic games: comparing trust game decisions to other social trust measures. Royal Society Open Science. 2022;9(9):210213.

8. Schäpermeier M, Thielmann I, Rau R. Which Measures of Beliefs About Others’ Prosociality Predict Prosocial Behavior in Economic Games? Collabra: Psychology. 2024;10(1).

9. Meuer M, Imhoff R. Believing in hidden plots is associated with decreased behavioral trust: Conspiracy belief as greater sensitivity to social threat or insensitivity towards its absence? J Exp Soc Psychol. 2021;93:104081.

10. Brülhart M, Usunier J-C. Does the trust game measure trust? Economics Letters. 2012;115(1):20-3.

11. Giancola PR, Parrott DJ. Further evidence for the validity of the Taylor Aggression Paradigm. Aggressive Behavior. 2008;34(2):214-29.

12. Chester DS, Lasko EN. Validating a Standardized Approach to the Taylor Aggression Paradigm. Soc Psychol Personal Sci. 2019;10(5):620-31.

13. West SJ, Hyatt CS, Miller JD, Chester DS. p-Curve analysis of the Taylor Aggression Paradigm: Estimating evidentiary value and statistical power across 50 years of research. Aggressive Behavior. 2021;47(2):183-93.

14. Payer DE, Lieberman MD, London ED. Neural Correlates of Affect Processing and Aggression in Methamphetamine Dependence. Arch Gen Psychiatry. 2011;68(3):271-82.

15. Zacher A, Zimmermann J, Cole DM, Friedli N, Opitz A, Baumgartner MR, et al. Chemical cousins with contrasting behavioural profiles: MDMA users and methamphetamine users differ in social-cognitive functions and aggression. Eur Neuropsychopharmacol. 2024;83:43-54.
